# Integrative analysis of the heat shock response in *Aspergillus fumigatus*

**DOI:** 10.1186/1471-2164-11-32

**Published:** 2010-01-15

**Authors:** Daniela Albrecht, Reinhard Guthke, Axel A Brakhage, Olaf Kniemeyer

**Affiliations:** 1 Research Group Systems Biology/Bioinformatics, Leibniz Institute for Natural Product Research and Infection Biology, Hans-Knöll-Institute, Jena, Germany; 2 Department of Molecular and Applied Microbiology, Leibniz Institute for Natural Product Research and Infection Biology, Hans-Knöll-Institute, Jena, Germany; 3 Friedrich Schiller University, Jena, Germany

## Abstract

**Background:**

*Aspergillus fumigatus *is a thermotolerant human-pathogenic mold and the most common cause of invasive aspergillosis (IA) in immunocompromised patients. Its predominance is based on several factors most of which are still unknown. The thermotolerance of *A. fumigatus *is one of the traits which have been assigned to pathogenicity. It allows the fungus to grow at temperatures up to and above that of a fevered human host. To elucidate the mechanisms of heat resistance, we analyzed the change of the *A. fumigatus *proteome during a temperature shift from 30°C to 48°C by 2D-fluorescence difference gel electrophoresis (DIGE). To improve 2D gel image analysis results, protein spot quantitation was optimized by missing value imputation and normalization. Differentially regulated proteins were compared to previously published transcriptome data of *A. fumigatus*. The study was augmented by bioinformatical analysis of transcription factor binding sites (TFBSs) in the promoter region of genes whose corresponding proteins were differentially regulated upon heat shock.

**Results:**

91 differentially regulated protein spots, representing 64 different proteins, were identified by mass spectrometry (MS). They showed a continuous up-, down- or an oscillating regulation. Many of the identified proteins were involved in protein folding (chaperones), oxidative stress response, signal transduction, transcription, translation, carbohydrate and nitrogen metabolism. A correlation between alteration of transcript levels and corresponding proteins was detected for half of the differentially regulated proteins. Interestingly, some previously undescribed putative targets for the heat shock regulator Hsf1 were identified. This provides evidence for Hsf1-dependent regulation of mannitol biosynthesis, translation, cytoskeletal dynamics and cell division in *A. fumigatus*. Furthermore, computational analysis of promoters revealed putative binding sites for an AP-2alpha-like transcription factor upstream of some heat shock induced genes. Until now, this factor has only been found in vertebrates.

**Conclusions:**

Our newly established DIGE data analysis workflow yields improved data quality and is widely applicable for other DIGE datasets. Our findings suggest that the heat shock response in *A. fumigatus *differs from already well-studied yeasts and other filamentous fungi.

## Background

*Aspergillus fumigatus *is a ubiquitous fungus that can be isolated from habitats such as soil or compost [1]. In the last decades, it has become the primary mold pathogen of humans. Especially in immunocompromised patients, the fungus is responsible for life-threatening infections. Such invasive aspergillosis (IA) is associated with a mortality rate of up to 90% [2]. The small size of spores permits ready access to the lung alveoli, which are the primary site of infection. *A. fumigatus *is highly thermotolerant, surviving temperatures of up to 70°C. Factors which convey thermo- and stress resistance at high temperature may also contribute to the virulence of this mold [3]. However, only few genes have been shown to be involved in thermotolerance of *A. fumigatus *so far and no direct link to virulence was found yet. A strain lacking the *o*-mannosyltransferase gene *Afpmt1 *was shown to be impaired in growth above 37°C [4] but did not show attenuation in virulence. A similar phenotype was shown for the thermotolerance protein ThtA, the function of which is unknown [5]. The nucleolar protein CgrA that is involved in ribosome biogenesis was shown to be essential for growth at 37°C *in vitro *and in infected mice, but was dispensable at 22°C [3]. Thermoresistance in *A. fumigatus*, therefore, is polygenic and mediated by numerous factors. For this reason, global studies are required to identify sets of transcripts or proteins, which are important for growth at elevated temperatures. Furthermore, a comparison of the heat shock response of *A. fumigatus *to that in other fungi may reveal differences that help to explain its thermotolerance.

The heat shock response of the budding yeast *Saccharomyces cerevisiae *is the best characterised both on the transcriptome and proteome level [6-12]. One of the primary effects of heat shock in this organism is un- and refolding of misfolded proteins which is mediated by heat shock proteins (HSPs, chaperones). Moreover, the expression of genes or proteins involved in cell wall integrity, cytoskeleton organisation, small molecular and vesicular transport, energy generation, defense against oxidative stress, signal transduction, carbohydrate metabolism, ubiquitination, and proteolysis are induced.

The heat and general stress response in yeast is regulated by the transcription factors Hsf1, Msn2/Msn4 and Hac1. The transcriptional regulator Hsf1 binds to specific repeat sequences (nGAAn or nTTCn) termed heat shock elements (HSE). Upon heat shock, Hsf1 induces the expression of chaperones and many other genes (reviewed in [13,14]). The second regulatory system of the heat shock response consists of Msn2 and Msn4. They play central roles in response to several stresses by activating gene expression via the stress response element (STRE) (reviewed in [13,7]). The third component is Hac1. This transcription factor activates the expression of genes the products of which promote protein folding in the endoplasmic reticulum (ER) and degradation of incorrectly folded proteins (reviewed in [15]).

In *A. fumigatus*, the global regulatory network induced upon heat shock has so far only been investigated on the transcriptome level [16-18]. To gain further insight into the heat shock response of *A. fumigatus *at the level of the proteome, we applied 2D-fluorescence difference gel electrophoresis (DIGE). This technique is superior to classical gel-based proteomics approaches. It enables separating different samples labelled with spectrally resolvable fluorescent dyes on the same 2D gel. This reduces experimental gel-to-gel variation and provides increased statistical confidence. However, raw DIGE data still reflect technical and biological variation. Additionally, missing intensity values may be a problem, depending on the software used for gel image analysis [19]. It was shown that the normalization methods of commercial software are not able to eliminate all bias and produce different results depending on the image analysis software used [20,21]. Therefore, several attempts have been made to reduce noise and to improve the quality of datasets by various statistical methods [19-21].

Here, we characterized the protein expression of *A. fumigatus *following a temperature shift from 30°C to 48°C. This rise in temperature induces a clear heat shock response in *A. fumigatus *and corresponds to temperature conditions in compost piles. Data analysis was optimized by imputing missing intensity values and subsequent data normalisation. Additionally, transcriptome and sequence information was used to achieve a comprehensive view of the *A. fumigatus *heat shock response. Furthermore, we compared our data with global studies of other fungi to elucidate possible factors which may distinguish a thermotolerant mold from mesophilic species. Our study revealed novel putative Hsf1-targets in *A. fumigatus*.

## Results

### Experimental Design

**Figure 1:**
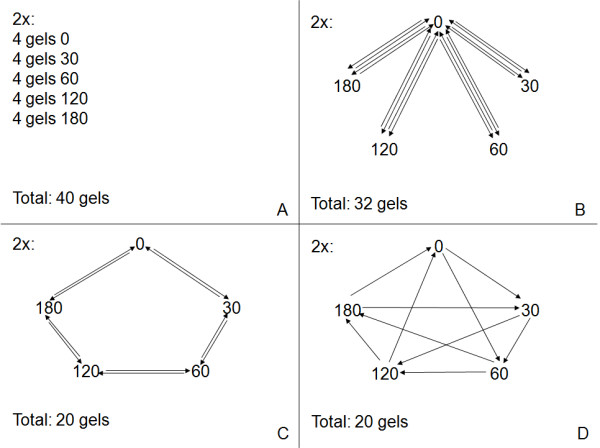
**Possible experimental designs for time series experiments**. 0, 30, 60, 120, and 180 each represent one biological sample taken at this time point (min.) after temperature shift; arrows point from the Cy3 labeled sample to the Cy5 labeled sample and represent gels; A -- non DIGE; B -- DIGE, reference design; C -- DIGE, loop design; D -- DIGE, extended loop design.

The DIGE technique has the advantage of increasing replicate number whilst keeping the number of 2D gels required relatively low. It allows multiplexing of up to three samples, labeled with spectrally resolvable fluorescent dyes (Cy3, Cy5, Cy2), in one gel [22]. The co-migration of proteins in one gel eliminates running differences between those samples. The additional use of a pooled internal standard results in better inter- and intra-gel matching of spots. It has been shown that this technique is able to generate statistically significant data with fewer 2D gels than traditional 2D gel electrophoresis [23]. Furthermore, the application of CyDyes ensures a linear detection over a wide range of protein abundance with around four orders of magnitude [24,25]. Applying the reference design (Figure 1B), as most studies do, results in the use of half of the gels for measuring the reference. Loop design (Figure 1C), is more suitable, because it reduces cost and experimental effort and, at the same time, it produces statistically sound data [26]. Extended loop design (Figure 1D) has the additional advantage of high intersample connectivity.

In this study, reference and extended loop design were applied. Loop design resulted in less missing data than reference design (31% vs. 46%). Therefore, more data could be used for further analyses (982 vs. 456 spots). This also resulted in the detection of more significant differentially regulated spots (183 vs. 55). Because of this, we focused on the data generated by application of the extended loop design.

### Image and data analysis

Imputation methods were selected from a set of described approaches which have previously been applied to proteomic datasets (e.g., [19,27-29]). The quality of these methods was assessed by several tests. The combination of minimal imputation and k-nearest neighbors was found to perform best [30]. It defines spots appearing in less than 25% of the gels of one experimental condition as noise. Present spot values from similarly regulated spots are used for imputing missing data of spots with less than 25% missing values across all gels. To our knowledge, the combination of these two methods was used and evaluated here for the first time and is also eligible for other DIGE studies.

The normalization procedure, using variance stabilization (vsn) first and local polynomial regression (loess) subsequently, was tested against no normalization, DeCyder standard normalization and normalization using only vsn or loess. The newly introduced combination of vsn and loess produced the best result in terms of noise reduction [31].

The whole data analysis workflow is publicly available as tool DIGE analyzer within the data warehouse OmniFung [32] at http://www.omnifung.hki-jena.de/Rpad/DIGE_analyzer.Rpad. Also, all experimental data produced in this study are stored in this data warehouse and are available by public login.

### Differentially regulated proteins at high temperature

Preliminary 2D-gel electrophoresis studies revealed only a small change in protein expression following an increase of the cultivation temperature from 30°C to 37°C (data not shown). Concordantly, transcriptome analysis of temperature shift experiments revealed a higher expression of heat-shock responsive genes following a shift from 30°C to 48°C in comparison to 37°C [17]. We therefore decided to focus our protein expression studies on the heat shock response at 48°C (representing temperature in compost). This also allows a better comparison of data from less thermotolerant, mesophilic fungi, such as *S. cerevisiae*, where a temperature of 37°C already induces a clear heat shock response.

**Figure 2:**
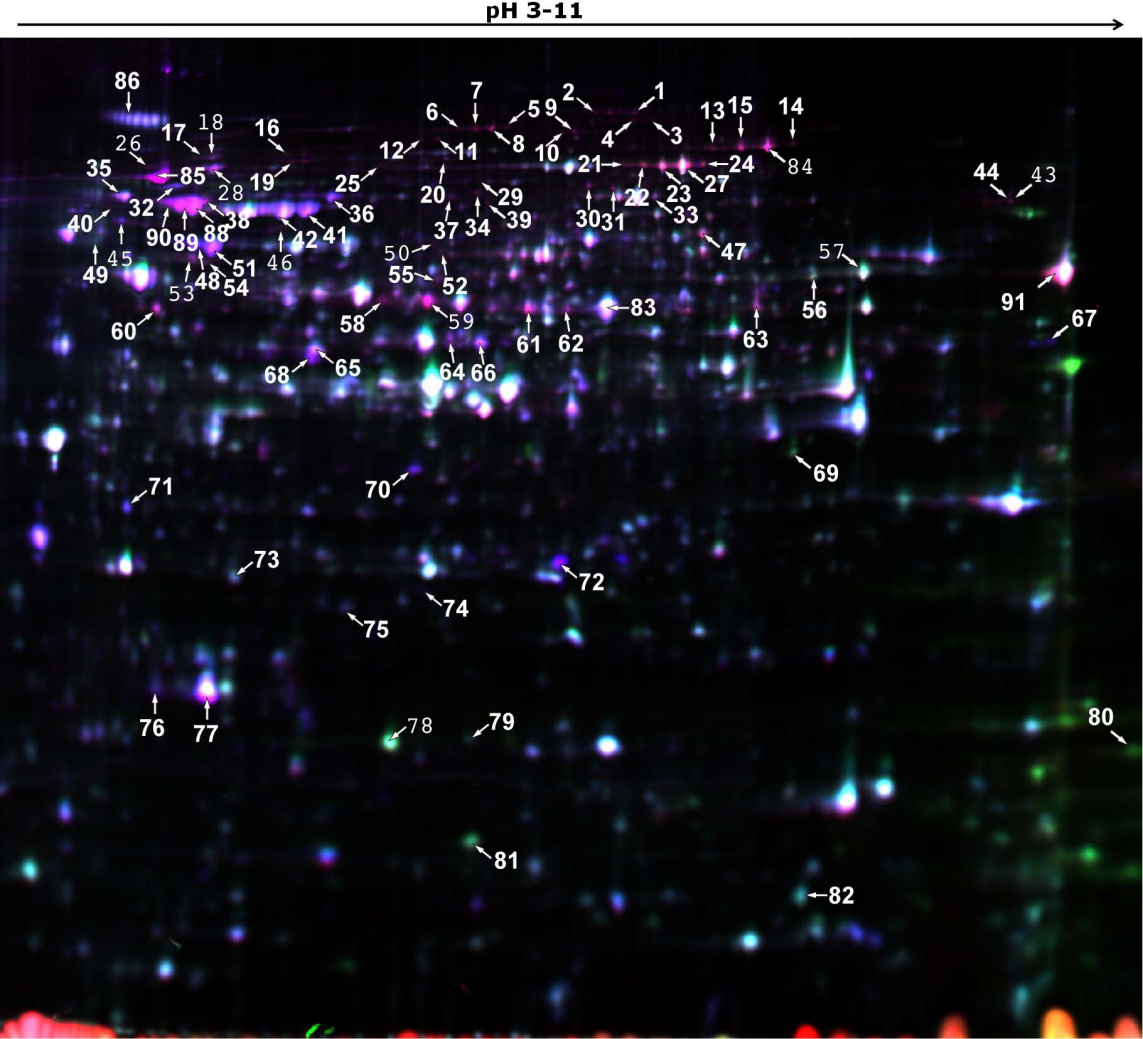
**2D-gel electrophoresis of protein extracts of *A. fumigatus *grown at 30°C (Cy3-blue colored) and 48°C (120 minutes after temperature shift; Cy5-green colored) including the pooled internal standard (Cy2-red colored)**. Proteins were stained with the difference in gel electrophoresis (DIGE) labeling technique. The orientation of the IEF is indicated and numbers refer to proteins whose levels changed significantly during growth at elevated temperature (see additional file 1, *MS_results.xls*, for protein information).

1886 spots were detected in the gels (Figure 2). A set of 183 spots was analyzed further. 91 of them, representing 64 different proteins, were identified by MS (additional file 1, *MS_results.xls*). The number of differently expressed proteins is similar to previous 2D-gel electrophoresis studies of the heat shock response in *S. cerevisiae *[10,33], *Penicillium marneffei *[34] and *Aspergillus flavus *[35]. All spots representing the same protein (up to five for some proteins) showed a very similar regulation. Therefore, the median value of these spots was calculated for each time point to define a protein as up- or downregulated. 54 proteins appeared to be upregulated, whereas 10 showed downregulation (Table 1). The putative functions identified for these proteins can be divided into several groups: many proteins have functions in protein folding (chaperones), in the organization of the cytoskeleton, transcription, translation and the oxidative stress response. The flavohemoprotein AFUA_4G03410 acts as an NO detoxification enzyme [36] and the nitroreductase family protein AFUA_5G09910 has a putative role in maintaining the cellular oxidative and/or nitrosative balance [37]. The regulatory subunit of the protein phosphatase 2A is presumably involved in hyphal growth [38] and in the repression of RNA-polymerase III transcription of rRNA and tRNA [39]. In addition, a large group of differentially regulated proteins was involved in amino acid biosynthesis and in carbohydrate metabolism: enzymes of glycolysis (phosphoglycerate kinase PgkA, hexokinase Kxk), TCA cycle (alpha-ketoglutarate dehydrogenase complex subunit Kgd1), pentose phosphate pathway (transketolase TktA; 6-phosphogluconate dehydrogenase Gnd1), mannose metabolism (mannitol-1-phosphare dehydrogenase), lipid biosynthesis (ATP citrate lyase subunit 1, acetyl coenzyme A synthetase FacA), and an NADP-dependent isocitrate dehydrogenase were affected by heat shock.

**Table 1:** Differentially regulated proteins in *A. fumigatus *after a temperature shift from 30°C to 48°C.

Protein	T = 30	T = 60	T = 120	T = 180	Osc
**Up regulated**					
***Chaperone***					
AFUA_1G07440 (molecular chaperone Hsp70)	1.95	2.45	2.06	2.45	No
AFUA_1G11180 (heat shock protein/chaperonin HSP78)	1.18	1.24	0.77	1.33	No
AFUA_1G12610 (HSP 70 chaperone HSP88)	1.19	1.06	0.59	1.34	Yes
AFUA_2G04620 (Hsp70 chaperone BiP/Kar2)	0.22	0.84	1.50	1.68	No
AFUA_2G09290 (antigenic mitochondrial protein HSP60)	0.43	1.28	2.17	1.88	No
AFUA_2G09960 (mitochondrial Hsp70 chaperone (Ssc70))	0.18	0.81	1.47	1.23	No
AFUA_3G14540 (heat shock protein Hsp30/Hsp42)	-0.24	1.23	2.37	2.35	No
AFUA_4G10010 (Hsp90 co-chaperone Cdc37)	1.13	1.22	1.84	1.97	No
AFUA_4G12850 (calnexin)	0.19	0.59	1.84	1.58	No
AFUA_5G04170 (molecular chaperone and allergen Mod-E/Hsp90/Hsp1)	3.11	3.68	3.13	3.76	No
AFUA_5G07340 (DnaJ domain protein Psi)	0.50	1.01	2.31	1.58	No
AFUA_7G01860 (heat shock protein (Sti1))	0.38	1.30	1.92	1.70	No
***Cell wall and cytoskeleton***					
AFUA_1G02550 (tubulin alpha-1 subunit)	1.04	0.92	-0.19	0.48	Yes
AFUA_2G07420 (actin-bundling protein Sac6)	1.48	1.27	0.30	1.16	Yes
***Transport***					
AFUA_6G07120 (nuclear movement protein NudC)	-0.64	0.60	1.79	1.18	Yes
***Energy generation***					
AFUA_2G13240 (V-type ATPase, B subunit)	0.95	0.66	0.56	1.02	No
***Defence against oxidative and nitrosative stress***					
AFUA_4G03410 (flavohemoprotein)	0.73	0.90	0.97	1.04	No
AFUA_4G09110 (cytochrome c peroxidase Ccp1)	0.00	1.29	1.92	2.03	No
AFUA_5G09910 (nitroreductase family protein)	-1.18	-0.53	1.02	1.32	No
AFUA_6G02280 (allergen Asp F3)	-0.54	0.35	1.62	1.13	Yes
***Signal transduction***					
AFUA_1G05610 (protein phosphatase 2a 65 kd regulatory subunit)	1.47	1.47	0.98	1.27	No
AFUA_4G12450 (conserved lysine-rich protein)	-0.01	0.21	1.34	1.09	No
***Carbohydrate metabolism***					
AFUA_1G10350 (phosphoglycerate kinase PgkA)	0.12	0.42	1.08	0.80	No
AFUA_1G13500 (transketolase TktA)	1.07	0.54	-0.62	-0.01	Yes
AFUA_2G05910 (hexokinase Kxk)	1.39	1.27	0.35	0.73	Yes
AFUA_2G10660 (mannitol-1-phosphate dehydrogenase)	1.70	1.67	1.09	1.46	No
AFUA_3G08660 (isocitrate dehydrogenase Idp1)	1.19	0.95	0.08	0.42	Yes
AFUA_4G04680 (FGGY-family carbohydrate kinase)	1.12	0.88	0.29	0.79	Yes
AFUA_4G11080 (acetyl-coenzyme A synthetase FacA)	1.64	1.27	-0.10	0.88	Yes
AFUA_4G11650 (alpha-ketoglutarate dehydrogenase complex subunit Kgd1)	1.72	1.39	0.49	0.95	Yes
AFUA_6G04210 (mannosyl-oligosaccharide glucosidase)	1.29	1.21	0.77	0.97	No
AFUA_6G04920 (NAD-dependent formate dehydrogenase AciA/Fdh)	1.17	0.66	-0.67	0.05	Yes
AFUA_6G08050 (6-phosphogluconate dehydrogenase Gnd1)	2.21	1.85	0.55	1.03	Yes
AFUA_6G10650 (ATP citrate lyase, subunit 1)	1.38	0.92	-0.16	0.33	Yes
***Nitrogen metabolism***					
AFUA_1G10130 (adenosylhomocysteinase)	1.88	1.46	0.05	0.60	Yes
AFUA_1G12840 (nitrite reductase NiiA)	2.75	2.30	1.19	1.54	Yes
AFUA_2G11260 (3-isopropylmalate dehydratase)	1.18	0.90	-0.09	0.44	Yes
AFUA_4G07360 (cobalamin-independent methionine synthase MetH/D)	1.53	0.66	-1.08	-0.08	Yes
AFUA_4G07710 (pyruvate carboxylase)	2.29	1.84	0.78	1.25	Yes
AFUA_4G10460 (homocitrate synthase)	1.56	1.04	0.31	0.88	Yes
AFUA_4G13120 (glutamine synthetase)	0.67	1.48	1.72	1.84	No
***Ubiquitination and proteolysis***					
AFUA_4G09030 (aminopeptidase)	1.58	1.31	0.51	1.30	Yes
AFUA_5G04330 (aminopeptidase)	1.07	1.08	0.07	0.35	Yes
***Protein biosynthesis/Translation***					
AFUA_1G02030 (eukaryotic translation initiation factor 3 subunit EifCb)	1.33	0.64	-0.10	0.43	Yes
AFUA_1G06390 (translation elongation factor EF-1 alpha subunit)	2.00	1.28	-0.51	0.69	Yes
AFUA_2G13530 (translation elongation factor EF-2 subunit)	1.88	1.48	-0.43	0.62	Yes
AFUA_3G08160 (eukaryotic translation initiation factor 4)	1.81	1.48	0.84	1.18	Yes
AFUA_4G12920 (histidyl-tRNA synthetase, mitochondrial precursor)	1.10	1.20	0.64	1.18	Yes
AFUA_5G05920 (glycyl-tRNA synthetase)	1.56	1.26	0.23	0.83	Yes
AFUA_6G04570 (translation elongation factor eEF-1 subunit gamma)	1.20	1.10	0.22	0.71	Yes
AFUA_7G05660 (translation elongation factor eEF-3)	2.02	1.43	-0.19	0.70	Yes
AFUA_8G03880 (alanyl-tRNA synthetase)	1.37	0.80	-0.22	0.32	Yes
***Transcription***					
AFUA_1G15620 (DEAD box RNA helicase HelA)	1.43	1.40	0.24	0.70	Yes
***Cell cycle***					
AFUA_2G17110 (cell division control protein Cdc48)	1.01	1.11	0.50	0.94	Yes
**Down regulated**					
***Chaperone***					
AFUA_2G13040 (mitochondrial co-chaperone GrpE)	-1.12	-0.73	0.21	-0.57	Yes
***Transport***					
AFUA_5G03690 (CRAL/TRIO domain protein)	-1.29	-0.61	0.37	-0.53	Yes
***Energy generation***					
AFUA_2G13010 (cytochrome c oxidase polypeptide vib)	-1.79	-1.54	-0.12	-1.12	Yes
***Carbohydrate metabolism***					
AFUA_1G09930 (glycerol dehydrogenase Gcy1)	-1.16	-1.44	-1.25	-0.98	No
AFUA_3G09230 (carboxylesterase)	0.14	-0.69	-1.07	-1.07	No
***Nitrogen metabolism***					
AFUA_3G09320 (serine hydroxymethyltransferase)	0.06	-0.67	-1.01	-1.12	No
***Protein biosynthesis/Translation***					
AFUA_6G02750 (nascent polypeptide-associated complex (NAC) subunit)	-1.35	-1.21	-0.85	-1.36	No
AFUA_6G12660 (40S ribosomal protein S10b)	-1.35	-1.21	-0.85	-1.36	No
***Transcription***					
AFUA_3G08580 (glycine-rich RNA-binding protein)	-1.27	-1.18	-0.84	-2.09	No
AFUA_8G05300 (RNA polymerase II subunit 7)	-1.38	-0.97	-0.72	-1.81	No

Relative protein abundance is depicted as log_2_-ratio. Logarithmized normalized intensities of the respective time points were subtracted by logarithmized normalized intensity of time point t = 0. Osc describes whether the protein is oscillatingly regulated.

**Figure 3:**
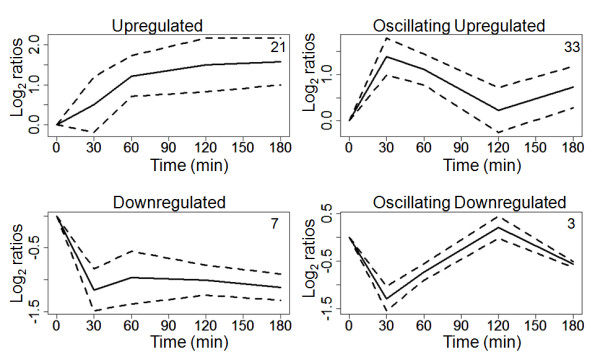
**Differentially regulated proteins in the heat response form four groups with similar time courses**. Solid lines represent median time courses and dashed lines the median absolute deviation of every time course group. Time is given in minutes and the relative abundance of protein spots in log_2 _ratios. In the right upper corner of every plot, the number of proteins in the group is displayed.

The 64 significant differentially regulated and identified proteins were grouped according to qualitative features of their time courses. In grouping, up- and downregulation was discriminated. Both groups of proteins were subdivided into oscillating and non-oscillating proteins. A protein was defined as being oscillating when the slope of its time course changed its algebraic sign twice, once at 30 or 60 minutes (first extremum) and once at 120 minutes (second extremum). Additionally, the difference between the log_2_-ratios of a spot at these time points had to be larger than the standard deviation of the log_2_-ratios from the whole time course of this spot (Figure 3). The grouping is in agreement with clusters calculated by fuzzy c-means algorithm (fcm) for the corresponding transcriptomic data [16]. Chaperones were mostly non-oscillating upregulated, as were proteins involved in defense against oxidative stress and signal transduction. Also, most of the downregulated proteins were non-oscillating. Proteins of the metabolism as well as of transcription and translation were upregulated in an oscillating manner.

In addition to the manual annotation of the differentially regulated proteins with a functional class as described above, FunCat categorization [40] was conducted. Most affected proteins have binding functions or need cofactors and are related to metabolism, cell rescue, defence and virulence or protein fate. Since the categories on the top level of FunCat are quite broad, an enrichment analysis was conducted on the second level. The following categories displayed a p-value below 0.05 (in descending order of significance): protein folding and stabilization, nucleotide binding, stress response, protein binding, animal cell type differentiation, translation, cell death, phosphate metabolism, assembly of protein complexes, glycolysis and gluconeogenesis, fungal/microorganismic development, tricarboxylic-acid pathway (citrate cycle, Krebs cycle, TCA cycle), aminoacyl-tRNA synthetases, cellular sensing and response, complex cofactor/cosubstrate binding, protein targeting, sorting and translocation, nucleus, and anaplerotic reactions. This enrichment shows that although many proteins belonged to the category metabolism, non-metabolic categories are more important in the process of heat shock. FunCat categories of different hierarchical levels for all differentially regulated proteins as well as all p-values of the enrichment test at level two can be found in additional file 2 (*funcat.xls*).

### Relation of transcriptome and proteome data

Whole genome analysis of only transcriptome or proteome data remains a reductionist approach, since both levels are strongly intertwined. To achieve a more holistic view, we analyzed previously published transcriptomic information. For half of the significant differentially regulated proteins the respective transcript was also found to be significantly regulated.

Low correlation of transcripts and corresponding proteins has been reported [41,42]. In this study, linear relationships were examined by Pearson correlation (PC) and non-linear relationships by Spearman correlation (SC). Median correlation for the whole time series of the 32 transcripts and respective proteins was -0.35 (PC) and -0.4 (SC). These values resulted from the fact that the expression of many proteins correlated negatively with their corresponding transcripts (see additional file 3, *time_series_tr_pr.doc*, for time course plots of transcripts and corresponding protein spots). PC ranged from -0.95 to 0.93, SC from -0.94 to 0.94. Both correlations depicted 22 of the 32 pairs of protein spots and transcripts showing negative values. Only 5 (PC, 4 SC) pairs showed good correlation (> 0.5). This means that most proteins seemed to be upregulated while their respective transcript was downregulated and vice versa. Since negative correlation between so many transcripts and their corresponding proteins is counterintuitive, a model of time delay was introduced. A protein is most probably not regulated at exactly the same time point as its transcript due to the fact that translation follows transcription. Therefore, the contradictory behavior of a protein in respect to its transcript can be the result of earlier events, before heat shock was applied. By analyzing time-shifted correlation between transcriptomic and respective proteomic data, it was found that different proteins showed different time delays (Table 2). A maximum delay of 105 minutes was allowed. Using this approach, all PC between a transcript and its respective protein became greater than 0.51 and the median correlation was good with a value of 0.84. Median correlation of SC was 0.89. Four SCs were below 0.5 (one remained below 0). For most proteins, the delay with the best correlation was the same for PC and SC. However, for eight proteins, there were differences of 15 minutes. This indicates that linear and non-linear dependencies between transcriptomic and proteomic data play a role. For one protein, a large difference of 105 minutes was calculated. This protein was the only one, where SC was below 0. Taken together, the following picture appeared: eight proteins responded in the first 30 minutes after transcript regulation (no or short delay). 18 proteins were 60 to 90 minutes delayed (medium delay), and five proteins responded 105 minutes after their transcript (strong delay). Proteins related to cell wall/cytoskeleton organization (tubulin-α Sac6), transport (NudC) and signal transduction (protein phosphatase 2a, conserved lysine rich protein) all responded with medium delay in respect to their transcript. Chaperones showed all different delays. Defense proteins against oxidative stress (allergen Asp F3, Ccp1) showed high delay. Carbohydrate metabolism (e.g., pentose phosphate shunt) and transcription proteins (RNA helicase HelA) responded with few or medium delay, while nitrogen metabolism and translation proteins responded with medium or strong delay.

**Table 2:** Comparison of the regulation on protein and transcript level.

Protein	PC of protein to transcript (no delay/best delay (best delay in minutes))	SC of protein to transcript (no delay/best delay (best delay in minutes))
***Chaperone***		
AFUA_1G07440 (molecular chaperone Hsp70)	0.48/0.52 (15)	0.26/0.52 (15)
AFUA_1G11180 (heat shock protein/chaperonin HSP78)	0.44/0.52 (15)	0.31/0.52 (15)
AFUA_1G12610 (HSP 70 chaperone HSP88)	0.72/0.72 (0)	0.60/0.60 (0)
AFUA_2G09290 (antigenic mitochondrial protein HSP60)	0.33/0.86 (75)	0.09/0.86 (75)
AFUA_2G13040 (mitochondrial co-chaperone GrpE)	0.80/0.80 (0)	0.83/0.83 (0)
AFUA_3G14540 (heat shock protein Hsp30/Hsp42)	-0.36/0.95 (105)	-0.51/0.89 (90)
AFUA_4G10010 (Hsp90 co-chaperone Cdc37)	0.18/0.76 (105)	-0.03/-0.03 (0)
AFUA_5G04170 (molecular chaperone and allergen Mod-E/Hsp90/Hsp1)	0.60/0.75 (15)	0.14/0.58 (30)
AFUA_5G07340 (DnaJ domain protein Psi)	-0.35/0.82 (75)	-0.09/0.96 (90)
AFUA_7G01860 (heat shock protein (Sti1))	0.02/0.9 (90)	-0.09/0.96 (90)
***Cell wall and cytoskeleton***		
AFUA_1G02550 (tubulin alpha-1 subunit)	-0.52/0.97 (60)	-0.43/0.98 (60)
***Transport***		
AFUA_5G03690 (CRAL/TRIO domain protein)	-0.73/0.78 (60)	-0.77/0.88 (75)
AFUA_6G07120 (nuclear movement protein NudC)	-0.24/0.68 (90)	-0.60/0.86 (90)
***Defence against oxidative and nitrosative stress***		
AFUA_4G09110 (cytochrome c peroxidase Ccp1)	0.76/0.94 (105)	0.60/0.94 (105)
AFUA_5G09910 (nitroreductase family protein)	-0.75/0.83 (105)	-0.71/0.43 (105)
***Signal transduction***		
AFUA_1G05610 (protein phosphotase 2a 65 kd regulatory subunit)	-0.60/0.88 (90)	-0.54/0.96 (90)
***Carbohydrate metabolism***		
AFUA_2G05910 (hexokinase Kxk)	-0.95/0.91 (90)	-0.83/0.96 (90)
AFUA_2G10660 (mannitol-1-phosphate dehydrogenase)	-0.13/0.51 (15)	0.03/0.57 (15)
AFUA_3G08660 (isocitrate dehydrogenase Idp1)	-0.76/0.91 (90)	-0.77/0.93 (90)
AFUA_4G11080 (acetyl-coenzyme A synthetase FacA)	-0.79/0.97 (90)	-0.71/0.93 (90)
AFUA_6G10650 (ATP citrate lyase, subunit 1)	-0.450/0.94 (60)	-0.54/0.97 (60)
***Nitrogen metabolism***		
AFUA_1G12840 (nitrite reductase NiiA)	-0.22/0.95 (30)	-0.20/0.83 (30)
AFUA_2G11260 (3-isopropylmalate dehydratase)	-0.34/0.93 (105)	-0.37/0.94 (105)
AFUA_2G13530 (bifunctional tryptophan synthase TRPB)	-0.85/0.85 (90)	-0.83/0.93 (75)
AFUA_4G07360 (cobalamin-independent methionine synthase MetH/D)	-0.83/0.81 (75)	-0.83/0.96 (90)
AFUA_4G07710 (pyruvate carboxylase)	-0.54/0.96 (90)	-0.49/0.93 (90)
AFUA_4G10460 (homocitrate synthase)	-0.73/0.86 (75)	-0.60/0.89 (90)
***Protein biosynthesis/Translation***		
AFUA_1G02030 (eukaryotic translation initiation factor 3 subunit EifCb)	-0.86/0.89 (75)	-0.89/0.88 (75)
AFUA_6G12660 (40S ribosomal protein S10b)	-0.23/0.93 (90)	0.09/0.86 (90)
AFUA_7G05660 (translation elongation factor eEF-3)	-0.17/0.70 (105)	-0.03/0.60 (105)
***Transcription***		
AFUA_1G15620 (DEAD box RNA helicase HelA)	-0.83/0.82 (75)	-0.94/0.86 (90)
AFUA_3G08580 (glycine-rich RNA-binding protein)	0.93/0.93 (0)	0.94/0.94 (0)

If a protein was present in more than one spot, median correlation and mutual information of all spots is given. PC = Pearson Correlation, SC = Spearman Correlation.

### Comparison to yeast

**Figure 4:**
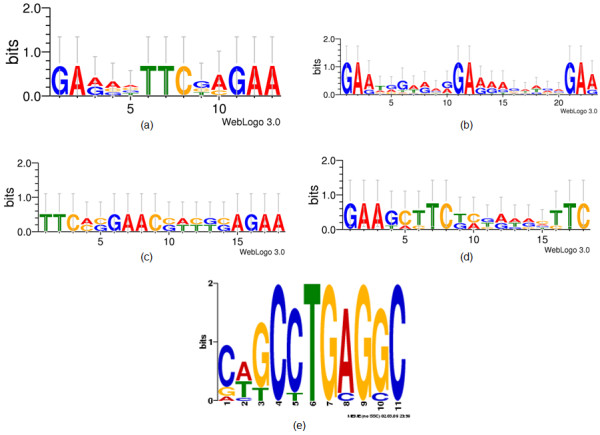
**Sequence logos for Hsf1 and AP-2alphaA binding motifs as found in the promoter region of genes whose proteins were differentially regulated upon heat shock**. Hsf: a (perfect), b (step), c (gap variant 1), d (gap variant 2), created with Weblogo, http://weblogo.threeplusone.com/; AP-2alphaA: e, created with MEME.

**Figure 5:**
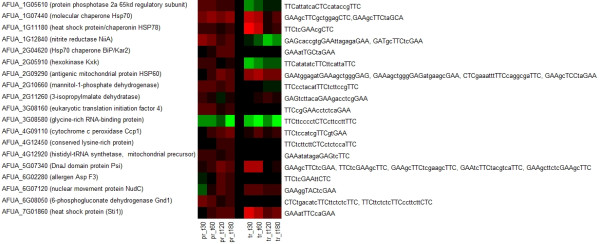
**Heatmap of potentially Hsf1 regulated proteins and transcripts**. Red color depicts upregulation, green color depicts downregulation. The gene/protein names are shown as well as the detected HSE.

In yeast, heat shock is largely governed by the transcription factors Hsf1, Msn2/4 and Hac1. Many targets of those have already been elucidated [11,12,14,43,44]. Hsf1 was very recently found to be upregulated in *A. fumigatus *under heat shock [18]. We looked for putative Hsf1 binding signatures [12,14] in the genome of *A. fumigatus*. By using ScanProsite [45], 17 genes with a potential heat shock element (HSE) in their promoter region were detected (see Figure 4 for motif logos and Figure 5 for heatmap of transcript and protein regulation). Proteins probably regulated by Hsf1 include chaperones (HSP70, HSP78, mitochondrial HSP60, Sti1), enzymes of the oxidative stress response (cytochrome C peroxidase Ccp1, allergen Asp F3), signal transduction (protein phosphotase 2a 65 kd regulatory subunit, conserved lysine rich protein), carbohydrate and nitrogen metabolism (Hexokinase Kxk, mannitol-1-phosphate dehydrogenase, 6-phosphogluconate dehydrogenase Gnd1, nitrite reductase NiiA, 3-isopropylmalate dehydrogenase), protein biosynthesis/translation (eukaryotic translation initiation factor 4, histidyl-tRNA synthetase, DNAJ domain protein Psi) and transcription (glycine-rich RNA-binding protein) (for explicit binding sites see additional file 4, *sequence_analysis.xls*). By using MEME [46], one additional chaperone (BiP/Kar2) and one transport protein (nuclear movement protein NudC) with slightly modified HSE motifs were found. Transcriptional activation of genes coding for chaperones is well known from yeast and higher organisms, but for some other genes (Ccp1, protein phosphotase 2a, conserves lysine rich protein, mannitol-1-phosphate dehydrogenase, Idp1, 3-isopropylmalae dehydrogenase, eukaryotic translation initiation factor 4, histidyl-tRNA synthetase, glycine-rich RNA-binding protein, NudC) regulation by Hsf1 has not been previously elucidated for yeast (for comparison with yeast homologues see additional file 4). For the transcription factors Msn2/4 and Hac1 neither ScanProsite nor MEME provided useful results, since the binding motifs are very short. In addition to the Hsf1 binding sites, MEME identified a possible binding motif for an AP-2alphaA-like transcription factor in 11 of the 64 differentially regulated proteins (AFUA_1G05610, AFUA_4G07710, AFUA_1G10130, AFUA_5G04170, AFUA_5G07340, AFUA_2G17110, AFUA_2G10660, AFUA_3G09320, AFUA_6G12660, AFUA_1G02030, AFUA_7G01860; see Figure 4 for motif logo). The AP-2 family of transcription factors regulates proliferation and differentiation during embryonic development in animals [47]. To date, no AP-2 homologues have been detected in any eukaryotic microorganism.

## Discussion

Proteome analysis based on the DIGE technique was used to explore how protein expression changes over time in response to a temperature shift from 30°C to 48°C. Data pre-processing methods (missing value imputation, normalization, filtering) were assessed and applied to improve the outcome of the image analysis.

The imputation and normalization approach used in this study combined previously described methods. Although each single method has been described before [21,28,48,49], the combination of them has not been used and validated for proteomics data so far. These methods can in principle be applied to any DIGE dataset by using the online tool DIGE analyzer.

Grouping of significant differentially regulated proteins resulted in two groups with straight up- or downregulation and two groups that depicted oscillatory behavior. This can be explained by feedback loops in the process of heat shock response. Since the absolute value of the second extremum was in both cases lower than the first one, the oscillations are damped and represent regulating oscillations, which are typical for biological systems after perturbation.

### Heat shock response of *A. fumigatus *characterized on the protein level

A sudden temperature change from 30°C to 48°C elicited a rapid alteration in protein expression and massive changes were already detectable after 30 minutes. The concurrent increase of HSPs of the cytoplasm, mitochondria and ER supports the general notion that heat shock is characterized by unfolding and disassembly of macromolecular structures, most notably proteins [50]. The relative abundance of most of the HSPs peaked at 120 minutes and only increased slightly thereafter or stayed at the same level. In this respect, the heat shock response of *A. fumigatus *does not differ significantly from that of yeast and other fungi. A transient increase of HSP transcripts within 60 minutes was also reported for *S. cerevisiae *and *Schizosaccharomyces pombe*. For these organisms, the maximum expression of HSP genes was detected at around 15 minutes [11,12,51]. HSP 30/HSP42 and HSP 90 showed the highest increase in abundance during the heat shock response of *A. fumigatus*. HSP90 is a highly abundant cytosolic chaperone. It is essential for the proper function of a diverse set of key regulators of growth, development and defense and is known to be induced upon heat shock [52]. It was also described as an allergenic protein of *A. fumigatus *[53]. The alpha-crystalline-type HSP30 protein is highly upregulated in many filamentous fungi upon heat shock [34,35,54]. It is presumably involved in the import of proteins to the mitochondrion [55] and was shown to be upregulated under oxidative stress conditions in *A. fumigatus *[56].

Additionally, enzymes of the oxidative and nitrosative stress response were induced upon heat shock in *A. fumigatus*. It is known from yeast, that heat shock enhances oxygen respiration. This results in an increase in the formation of reactive oxygen intermediates (ROI) and an activation of the oxidative stress response [57]. The data indicate a putative heat shock-dependent upregulation of the thioredoxin peroxidase AspF3 and the cytochrome c peroxidase Ccp1. An upregulation of enzymes involved in the depletion of reactive nitrogen intermediates (RNI) upon heat shock has not been previously described for fungi. At higher temperature, higher amounts of reactive nitrogen intermediates may be endogenously generated, in particular during growth with nitrate as sole nitrogen source. Nitrate has to be assimilatory reduced to ammonium, a process during which RNI can be produced.

Interestingly, many differentially regulated proteins were involved in carbohydrate and nitrogen metabolism. Most of these enzymes showed an oscillatory upregulation with a maximum at 120 minutes. An increase in the abundance of proteins involved in glycolysis could provide energy needed for the ATP-dependent protein refolding by chaperones [11]. A heat shock-dependent regulation of the glycolytic enzymes phosphoglycerate kinase and hexokinase was also demonstrated for other fungi [6,34,58].

In addition, enzymes of the NADPH-generating pentose phosphate pathway were upregulated. They are probably involved in balancing the redox state of *A. fumigatus *after heat stress by providing reducing equivalents for the reduction of ROI or oxidized glutathion or thioredoxin. The glucose-6-phosphate dehydrogenase was shown to be implicated in the adaptive response to oxidative stress in *S. cerevisiae *[59], but also showed a heat shock-dependent regulation in yeast [9]. Heat shock may also lead to increased fatty acid biosynthesis activity due to membrane lipid damage. The cytosolic ATP-citrate lyase and the acetyl coenzyme A synthetase were upregulated after heat shock on the protein level [60]. These enzymes can provide acetyl-CoA as precursor for the biosynthesis of fatty acids.

Nitrogen metabolism was also affected by the temperature shift. Heat shock leads to an increased turnover of proteins and hence to a higher rate of amino acid biosynthesis in *A. fumigatus*. This is reflected by the increased levels of enzymes involved in the biosynthesis of amino acids. In contrast, amino acid synthesis is repressed in *S. cerevisiae *after heat shock [11,61]. Inconsistent to this regulation, many transcripts of amino acid biosynthesis genes showed a converse regulation in comparison to their corresponding protein in *A. fumigatus*. This phenomenon can best be explained by post-transcriptional regulation such as phosphorylation or acetylation.

Recently, a state space model was used to examine the regulation of heat shock and metabolism genes [18]. The study found a negative association of many HSPs and their regulated metabolic genes. It was most prominent in the temperature shift from 30°C to 37°C and much weaker at 48°C. At the proteomic level, even this weak negative association cannot be confirmed, since all metabolic proteins showed a similar time course as the HSPs.

### Relation of transcriptome and proteome data

Comparison of proteome and transcriptome data was not possible on a genome-wide scale. Only 32 proteins and respective transcripts were detected as differentially regulated in both datasets. Therefore, we analysed the correlation of each pair of transcript and protein separately, instead of looking for global relations. When the proteome data were compared with the transcript data of Nierman *et al*. [17], a low correlation for many transcripts and their corresponding proteins was found. Similar observations were made during a genome-wide analysis of the effect of heat shock on an *S. cerevisiae *mutant strain [33]. However, the transcript level of some genes, e.g. HSPs, correlated well with the corresponding protein levels. This was particularly true when a time delay between transcription and translation was taken into account. Part of this inconsistent regulation of transcripts and proteins is explainable by known regulation mechanisms. (i) In a wide variety of eukaryotes, many stress conditions including heat stress lead to a repression of translation initiation and an accumulation of translationally repressed mRNA in either so-called stress granules or in the nucleus. Under these conditions, mRNA is stabilized by the inhibition of poly(A) shortening, because deadenylation normally leads to an increased mRNA decay [62]. (ii) Under conditions of heat shock, mRNAs of HSP genes are selectively exported from the nucleus for protein biosynthesis, whereas the bulky poly(A) mRNA remains in the nucleus [63]. However, it cannot be ruled out that the different *A. fumigatus *strains and media used by us (AMM) and Nierman *et al *(complete media) caused a difference in gene and protein expression.

For the comparison of proteomic and transcriptomic data, two correlation measures were used. Both are qualitative measures. They result in only one value for each transcript and protein pair. In the future, it would also be interesting to use methods which provide quantitative information for the comparison of data from both cellular levels.

### Putative Hsf binding sequences in the promoter region of heat-shock regulated proteins

The target genes of the transcriptional regulator Hsf1 contain a *cis*-acting sequence, the heat shock element (HSE). Using ScanProSite, 24 HSEs in promoter regions of 17 putative target genes of Hsf1 were identified in *A. fumigatus*. Using MEME, 5 additional motifs and 2 further putative target genes were identified. They had at most one modification that has not been reported in the literature. All three known HSE motifs (perfect, gap and step type, see additional file 4 for motif consensus sequences) were present in the promoter region of heat-shock induced genes. Among the putatively Hsf-regulated genes (Figure 5), many known Hsf-targets were found such as HSPs, a thioredoxin peroxidase (AspF3), enzymes of the pentosephosphate shunt (6-phosphogluconate dehydrogenase) and glycolysis (hexokinase) [9,58]. However, other *A. fumigatus *genes with an HSE-element have so far not been associated with Hsf-regulation. In yeast, the cytochrome c peroxidase was shown to be regulated by the Msn2/Msn4-dependent general stress response [7], but in *A. fumigatus *its expression is presumably induced by Hsf1. The cytochrome c peroxidase degrades ROI in mitochondria and is involved in conveying the oxidative stress signal [64].

Hsf1 may also be a regulator of tRNA/rRNA transcription (protein phosphatase 2a, histidyl-tRNA synthetase, glycine-rich RNA binding protein), translation (eukaryotic translation initiation factor 4) and leucine biosynthesis (3-isopropylmalate dehydratase). Interestingly, the mannitol-1-phosphate dehydrogenase is also a potential target of Hsf1 in *A. fumigatus*. It is the first enzyme in the pathway for the biosynthesis of mannitol, which appears to be essential for the protection against various stress conditions including heat as shown in *A. niger *[65].

The protein NudC is involved in nuclear migration during mitosis and hyphal tip growth in *A. nidulans*, but also implicated in fungal cell wall biosynthesis [66]. Under heat stress conditions, the transcription of the NudC gene may be under transcriptional control of Hsf1 to maintain cytokinesis and cell proliferation at higher temperature. Interestingly, the NudC orthologue NUD-1 in the nematode *Caenorhabditis elegans *exhibits chaperone activity [67].

Of the aforementioned putatively Hsf1 regulated genes, mannitol-1-phosphate DH, cytochrome c peroxidase and NudC showed the most typical Hsf1-driven rapid upregulation on the transcript level and a corresponding increase of the protein product (Figure 5). Interestingly, in the promoter region of the orthologous genes in *A. flavus*, *A. nidulans *and *A. terreus*, an HSE-sequence was not detectable. Further studies are needed to verify whether this regulation is unique to *A. fumigatus *and whether it could contribute to the observed thermotolerance in this fungus.

Furthermore, the relevance of the AP-2α-like binding motif in the promoter region of some heat-induced genes has to be validated experimentally. Homologues of AP-2 like transcription factors have not been found in fungal genomes yet.

## Conclusions

In our proteome study, the heat shock response of *A. fumigatus *was analyzed. For this purpose, a workflow was established that includes missing value imputation, data normalization and filtering of differentially regulated proteins. The workflow is shown in additional file 5 (*workflow.tif*). This workflow could be applicable to many DIGE datasets. Therefore, the described tool is publicly available in the internet.

The analysis of the adaptation of *A. fumigatus *to high temperatures revealed many similarities but also some obvious differences to the heat shock response compared to well-studied, mesophilic fungi such as *S. cerevisiae *and *S. pombe*, which are not able to grow at temperatures beyond 40°C.

The heat shock response in *A. fumigatus *was transient and most changes in protein expression appeared within two hours. Afterwards, the level of protein expression continued on a different, often higher level (e. g. HSPs) or dropped to the initial level (e.g. glycolytic enzymes). Besides the well known increased biosynthesis of HSPs upon heat stress, several other processes were influenced in *A. fumigatus *during heat shock: oxidative stress response, signal transduction, transcription, translation, energy generation, carbohydrate and nitrogen metabolism and cytoskeleton organisation. Additionally to the Hsf1 regulon, other stress regulators are most likely involved in the heat shock response of *A. fumigatus*.

Novel potential Hsf1 targets were identified. They seem to be *A. fumigatus *specific and mediate oxidative stress resistance in mitochondria, function as osmoregulator or ROI scavenger or are required for nuclear migration.

It is interesting to speculate that some of the putatively Hsf1-regulated target proteins described here mediate the high thermotolerance of *A. fumigatus*.

## Methods

### Experimental Design

In this study, the fungal response to heat shock at the time points 0, 30, 60, 120, and 180 minutes after a shift from 30°C to 48°C was analyzed. These time points were chosen to enable a good comparison with recent transcriptomic temperature shift data from *A. fumigatus *[17]. The temperature of 48°C was chosen to induce a clear heat shock response with an induction of heat shock responsive genes in the thermotolerant fungus [17].

An extended loop design [26] was applied, using two biological replicates which included four technical replicates in dye swap (Figure 1D). This design allows creating eight replicates for each of the five time points by using only 20 DIGE gels.

### Strains and culture condition

*Aspergillus fumigatus *wild-type strain ATCC 46645 was cultivated in *Aspergillus *minimal medium (AMM) with glucose as sole carbon and energy source as described in Weidner *et al*. [68]. Flasks containing 100 ml AMM were inoculated to a concentration of 3 × 10^6^conidia/ml and incubated overnight on a rotary shaker with 200 rpm at 30°C. After 16 hours, heat shock was induced by cultivating part of the cultures for four hours at 48°C. Control cultures were kept at 30°C. Mycelium was harvested by filtering the culture through Miracloth (Calbiochem, Germany). Subsequently, the mycelium was rinsed with demineralized water, pressed to remove liquid, frozen in liquid nitrogen, and stored at -70°C.

### DIGE 2D-gel electrophoresis

Samples for two-dimensional polyacrylamide gel electrophoresis (2D-PAGE) were prepared as previously described [56,69]. Briefly, frozen mycelium was ground in a pre-cooled mortar with a pestle in the presence of liquid nitrogen. Proteins of the crude extract were precipitated by TCA/acetone as described in Kniemeyer *et al*. [69]. The air-dried pellet was dissolved in lysis buffer containing 7 M urea, 2 M thiourea, 2% (w/v) CHAPS, 1% (w/v) Zwittergent 3-10, and 30 mM Tris. The pH of the sample was adjusted to 8.5 by addition of 100 mM NaOH. Protein concentration was determined according to the method of Bradford [70] using BIO-RAD protein assay (BIO-RAD Lab., Hartfordshire, USA). Afterwards, 50 μg protein samples were labeled with 30 pmol of CyDyes according to Lessing *et al*. [56]

Rehydrated IPGstrips (7 M urea, 2 M thiourea, 2% [w/v] CHAPS, 1% [w/v] Zwittergent 3-10, 0.002% [w/v] bromophenol blue, 0.5% [vol/vol] IPG buffer 3-11, 1.2% [vol/vol] De-Streak reagent) of 24 cm covering a nonlinear pH range from 3-11 were used for isoelectric focusing. 150 μg protein of pooled, mixed samples and in addition 100 μg unlabelled protein mixture were loaded via anodic cup loading onto IPGstrips. Isoelectric focusing was carried out as described previously [69]. Equilibrated IPGstrips were placed on 12.5% polyacrylamide gels, fixed with melted agarose and separated using an Ettan DALTsix electrophoresis system. Protein spots were visualized by a Typhoon 9410 scanner (GE Healthcare Biosciences) using a resolution of 100 μm. Images were cropped and spots were detected and quantified with the DeCyder 6.5 software package (GE Healthcare Biosciences). All proteins were subsequently visualized by Colloidal Coomassie staining as described by Neuhoff *et al*. [71].

### Protein identification by MS

Significantly regulated proteins were excised and tryptically digested according to the protocol of Shevchenko *et al *[72]. Peptides were extracted for one hour with acetonitrile (ACN): trifluoracetic acid (TFA) 0.1% (1:1 v/v), mixed with saturated a-cyano-4-hydroxycinnamic acid in ACN:TFA 0.1% (1:2 v/v) and allowed to dry on an MTP 800/384 anchor chip target (Bruker Daltonics, Germany). The samples were measured on an Ultraflex I MALDI-TOF/TOF device using flexControl 3.0 for data collection and flexAnalysis 3.0 (Bruker Daltonics GmbH, Germany) for spectra analysis/peak list generation as described in Vödisch *et al*. [73]. Up to five peptides of the peptide mass fingerprint (PMF) spectra were chosen for post source decay MS/MS analyses. For identification, peptide mass fingerprint (PMF) and peptide fragmentation fingerprint (PFF) spectra were submitted to the MASCOT server (MASCOT 2.1.02, Matrix Science, UK), searching the taxon fungi in the NCBI database. With respect to the sample preparation, fixed modification of cysteins to S-carbamidomethyl derivatives and variable methionine oxidation was defined for the database search. Further, no missed cleavage, and a peptide mass tolerance of 50 ppm was allowed. Results were regarded as significant with an allowed likelihood for a random hit of p = 0.05, according to the MASCOT score. Database searches were triggered and archived on a Proteinscape 1.3 database server (Protagen, Germany). Accuracy of raw peak lists was improved by automated internal recalibration using known contaminants (trypsin and keratin fragments) and application of the peak rejection filter of the Score Booster tool, implemented into the Proteinscape 1.3 database software.

### Image and data analysis

Quantification of protein expression was carried out with DeCyder 6.5 and resulted in 1886 spots. Raw data were exported out of DeCyder and analysis was done using the statistical software R (version 2.8.0 using Bioconductor packages impute, vsn, limma and sma).

In general, not all spots can be found on all gels by DeCyder and therefore show missing intensity values. In a first step, missing intensity values were imputed. Spots which were absent in six or seven of the eight replicates of one condition were considered as absent proteins in this condition (and thus, the one or two present signals as noise). They were imputed using a Gaussian distribution as described by Chich *et al*. [28] with the minimal detected value of all gels as mean m and a variance of s^2 ^= m/3. This assures very low, but positive, intensity values. They are a little different from each other to preserve experimental variation for those spots. Spots which then had missing values in at most 25% of all gels (across all replicates) were imputed by k-nearest neighbor (knn) method as proposed by Jung *et al *[48]. All spots which still had missing values after this procedure were discarded, leaving 982 spots for analysis.

Second, data were normalized by variance stabilization (vsn) followed by local polynomial regression (loess) as described previously [31]. Additionally, after vsn and before loess, the internal Cy2 standard was used to remove gel specific differences. Subsequently, for each spot the median of intensities of all gels at the same time point was calculated. Median intensities of each time point were then divided by median intensity of time point t = 0 and logarithmized.

Log_2_-ratios of normalized spot intensities were filtered for differential regulation as described before [31] using Z-Scores and ANOVA. Z-Scores for each spot were calculated for each time point separately. All spots with at least one Z-Score outside the range of (-1.96, 1.96), representing the 95% confidence level, were regarded as being differentially regulated. This resulted in spots with log_2_-ratios outside the range of (-1.070, 1.020), representing (-2.099, 2.028) as fold change thresholds. Additionally, ANOVA p-values (corrected for multiple testing by the method of Benjamini and Hochberg [74]) of smaller than 0.05 were used as indicators for statistical significance. Spots were regarded as being significantly upregulated when at least one log_2_-ratio (between one time point and time point 0) was above 1.020, the maximal log_2_-ratio was larger than the absolute value of the minimal one (in case, a spot was first up- and then downregulated or vice versa) and when they showed an ANOVA p-value smaller than 0.05. Spots were regarded as being significantly downregulated when at least one log_2_-ratio was below -1.070, the absolute value of the minimal log_2_-ratio was larger than the maximal one and when they showed an ANOVA p-value smaller than 0.05.

Identified significant differentially regulated proteins were categorized according to the Functional Catalogue (FunCat [40]). FunCat information was taken from the PEDANT server of Munich Information Center for Protein Sequences [75]. First, top level categories were analyzed to get a broad overview of the functional categories. Second, an enrichment analysis using Fisher's exact test with all known categories for the whole proteome as reference set was conducted.

### Comparison to transcriptomic data

Pre-processed transcriptomic data, published in 2005 [17], were obtained from ArrayExpress [76] with accession number E-MEXP-332. For half of the differentially regulated proteins, the respective transcripts were also differentially regulated. Pearson (PC) and Spearman correlation (SC) for pairs of transcripts and respective proteins were calculated to depict relationships. Both were also calculated for time shifted proteomic data to find possible delays in translational regulation with respect to transcriptional regulation. Therefore, time points were interpolated every 15 minutes by means of adjacent time points.

### Comparison to yeast

NCBI ProteinBlast was used to find homologues of the differentially regulated proteins of this study in *S. cerevisiae*. ScanProsite [45] was applied to search for the well known transcription factor binding sites (TFBSs) of Hsf1, Msn2/4 and Hac1 from *S. cerevisiae *[11,12,14,43,44] in the differentially regulated proteins of *A. fumigatus*. Input were intergenic regions comprising up to 1000 bp upstream of the transcript (less in case that another transcript was located less that 1000 bp away from the analyzed one). Sequences were retrieved from CADRE [77] and adjusted. The proteins with detected TFBSs and the yeast homologues with known binding TFBSs were used as input in MEME [46] to find more proteins with little substitutions in the binding site. Additionally, promoters of all significant differentially regulated proteins were used to find additional, new motifs.

## Authors' contributions

DA participated in the design of the study, carried out all bioinformatical analyses, and drafted the manuscript. RG and AAB initiated the study, participated in designing the experiments and writing the paper. OK carried out the experimental work, participated in the design of the study and took part in drafting the manuscript. All authors read and approved the final manuscript.

## Supplementary Material

Additional file 1 **MS_results**. Results of the MALDI-TOF/TOF analysis of protein spots with significant change in abundance after heat shock. Click here for file

Additional file 2 **Funcat**. FunCat annotations for all 64 differentially regulated proteins on most detailed (sheet1), second general (sheet2) and most general level (sheet4), including enrichment analysis of second level annotations (sheet3). Click here for file

Additional file 3 **Time_series_tr_pr**. Graphical display of the time series data of differentially expressed transcripts and proteins. Only significantly regulated proteins/transcripts are depicted. Time is given in minutes and the relative abundance of protein spots in log_2 _ratios. Each pair of a transcript and its respective protein is displayed in a separate plot. Transcripts are depicted in red, proteins in blue. If there were several spots representing the same protein, several blue lines were drawn. Click here for file

Additional file 4 S**equence_analysis**. Detected Hsf1 binding motifs in the upstream region of the genes representing the differentially regulated proteins (sheet1) and yeast homologues with their regulation (sheet2). Click here for file

Additional file 5 **Workflow**. The DIGE analysis workflow of this study. Click here for file
