# Supplementary material for: Integrative analysis of the heat shock response in Aspergillus fumigatus
Source: BMC Genomics. 2010 Jan 15;11:32. doi: 10.1186/1471-2164-11-32 (PMC2820008; doi:10.1186/1471-2164-11-32)
Supplement: Additional file 3 — Time_series_tr_pr. Graphical display of the time series data of differentially expressed transcripts and proteins. Only significantly regulated proteins/transcripts are depicted. Time is given in minutes and the relative abundance of protein spots in log2 ratios. Each pair of a transcript and its respective protein is displayed in a separate plot. Transcripts are depicted in red, proteins in blue. If there were several spots representing the same protein, several blue lines were drawn. [file 1471-2164-11-32-S3.DOC]

| 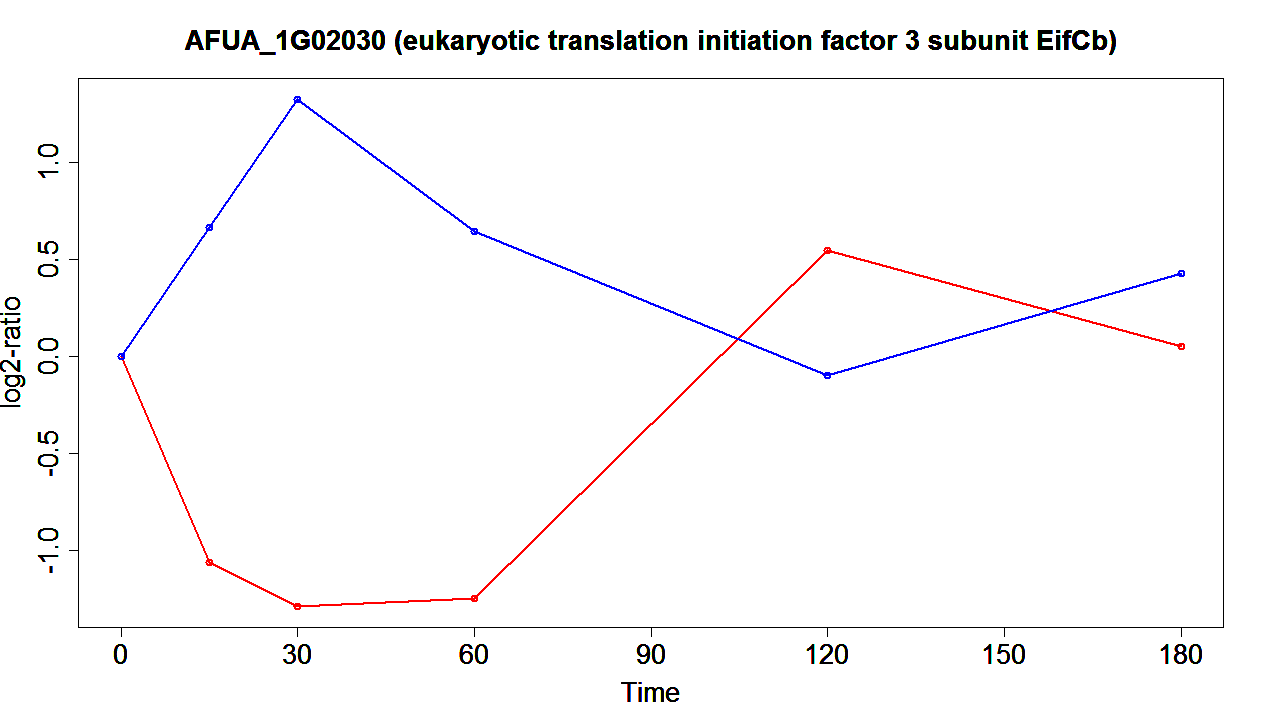  **Time series of differentially regulated transcripts and respective proteins**  **Relative abundance of protein spots in log_2_ ratios**  **Time in minutes after temperature shift**  **Red – transcript, blue – protein** |
| --- |
| 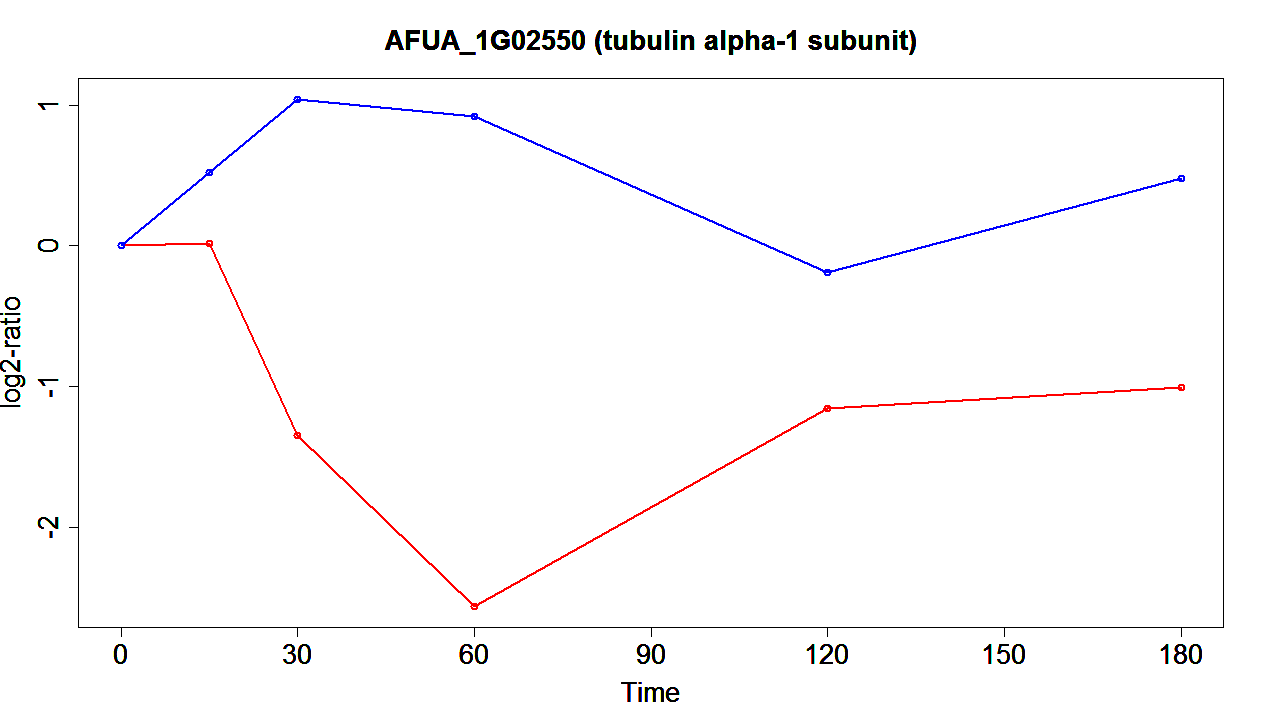  **Time series of differentially regulated transcripts and respective proteins**  **Relative abundance of protein spots in log_2_ ratios**  **Time in minutes after temperature shift**  **Red – transcript, blue – protein** |
| 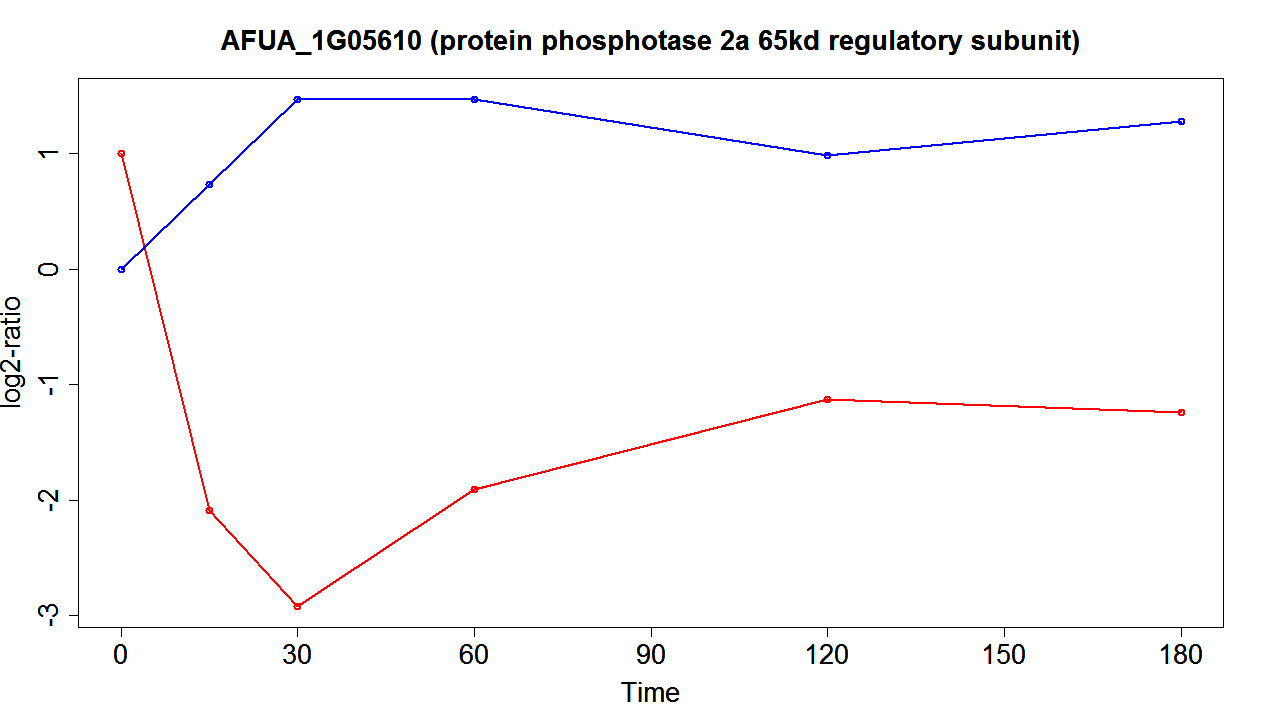  **Time series of differentially regulated transcripts and respective proteins**  **Relative abundance of protein spots in log_2_ ratios**  **Time in minutes after temperature shift**  **Red – transcript, blue – protein** |
| 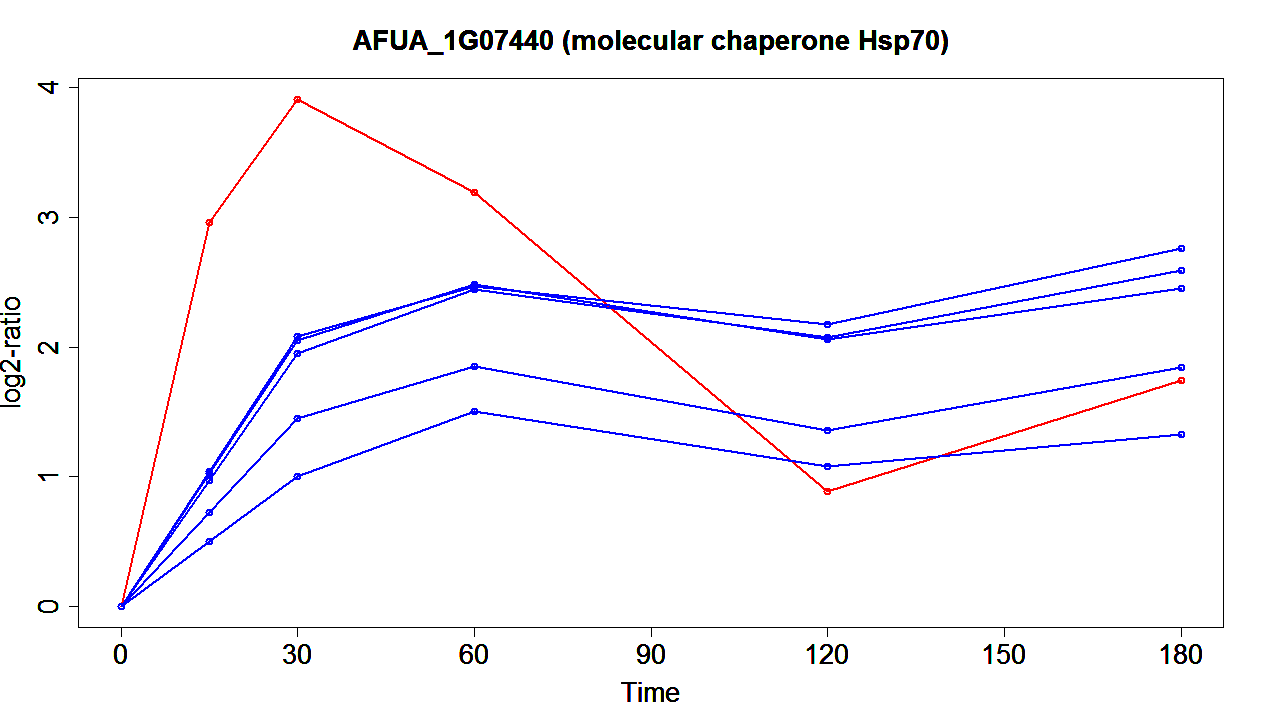  **Time series of differentially regulated transcripts and respective proteins**  **Relative abundance of protein spots in log_2_ ratios**  **Time in minutes after temperature shift**  **Red – transcript, blue – protein**  **several blue lines indicate several spots representing the same protein** |
| 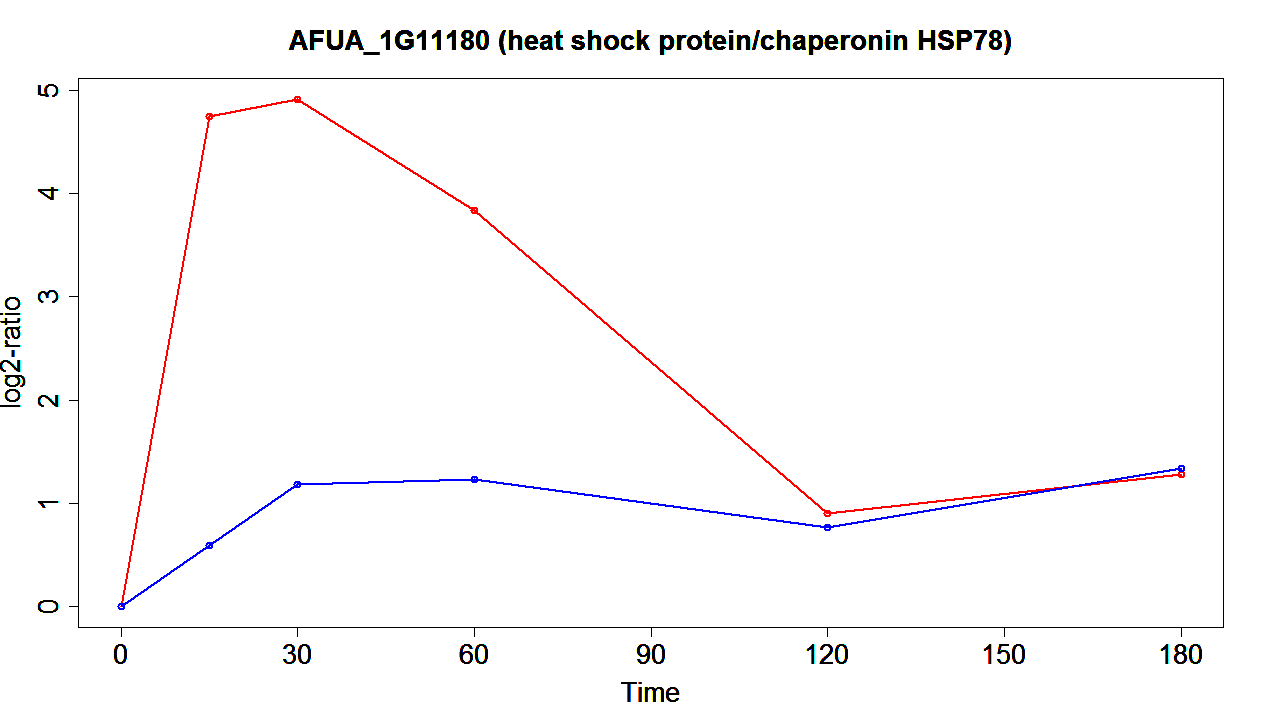  **Time series of differentially regulated transcripts and respective proteins**  **Relative abundance of protein spots in log_2_ ratios**  **Time in minutes after temperature shift**  **Red – transcript, blue – protein** |
| 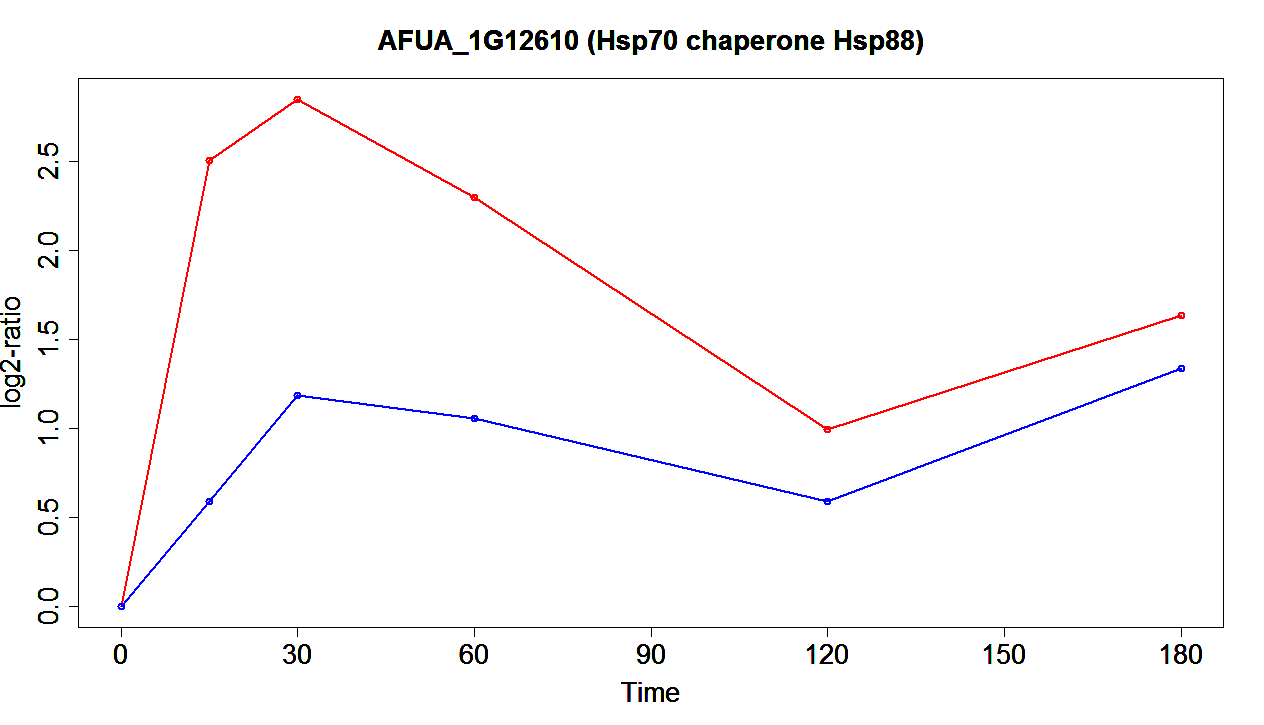  **Time series of differentially regulated transcripts and respective proteins**  **Relative abundance of protein spots in log_2_ ratios**  **Time in minutes after temperature shift**  **Red – transcript, blue – protein** |
| 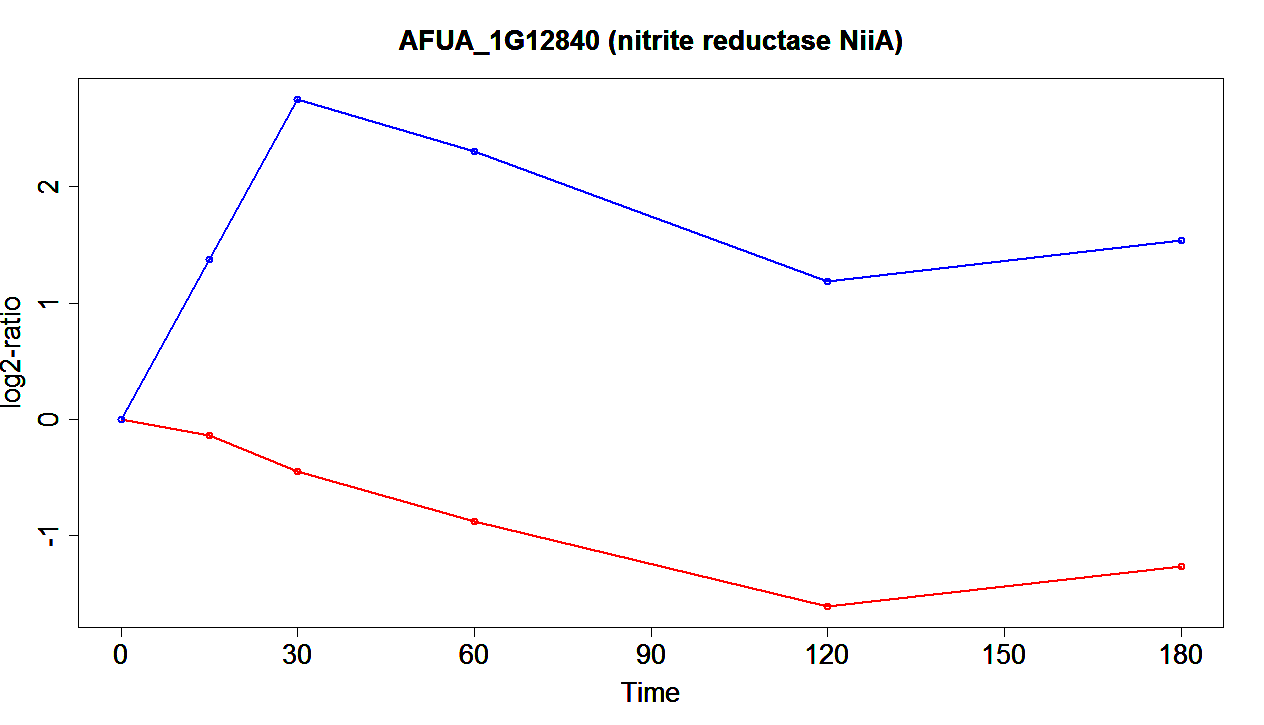  **Time series of differentially regulated transcripts and respective proteins**  **Relative abundance of protein spots in log_2_ ratios**  **Time in minutes after temperature shift**  **Red – transcript, blue – protein** |
| 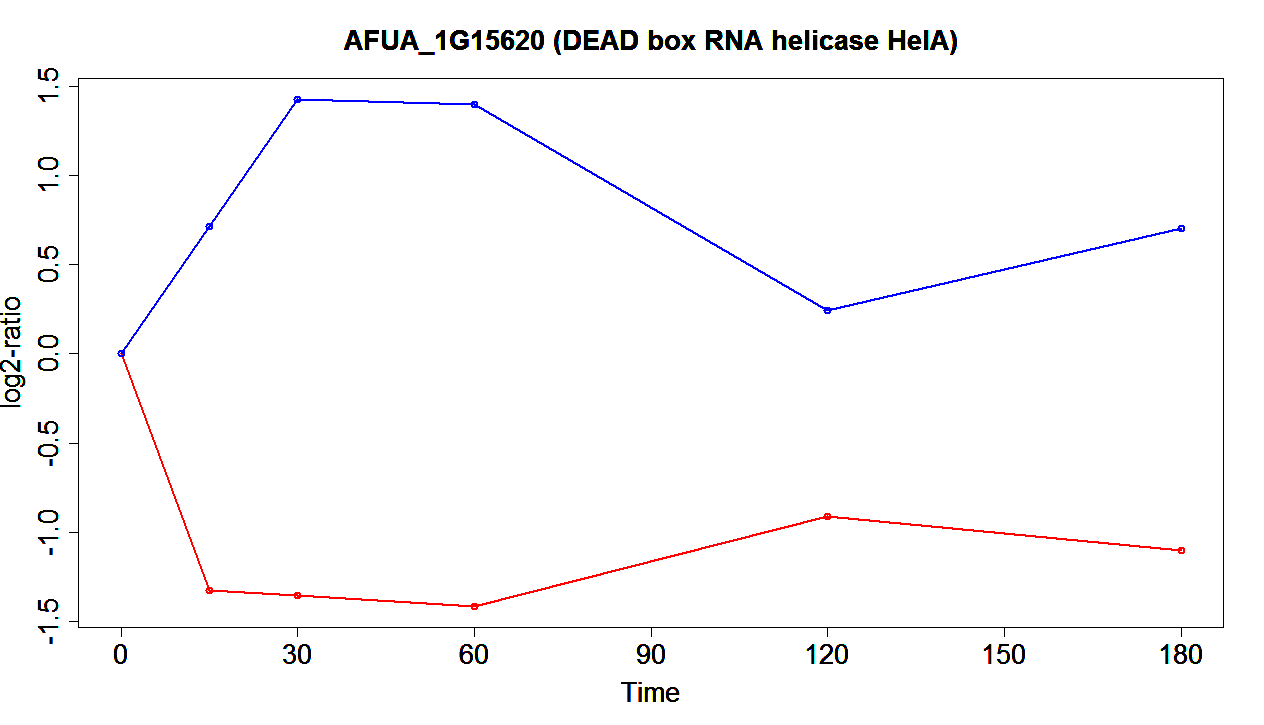  **Time series of differentially regulated transcripts and respective proteins**  **Relative abundance of protein spots in log_2_ ratios**  **Time in minutes after temperature shift**  **Red – transcript, blue – protein** |
| 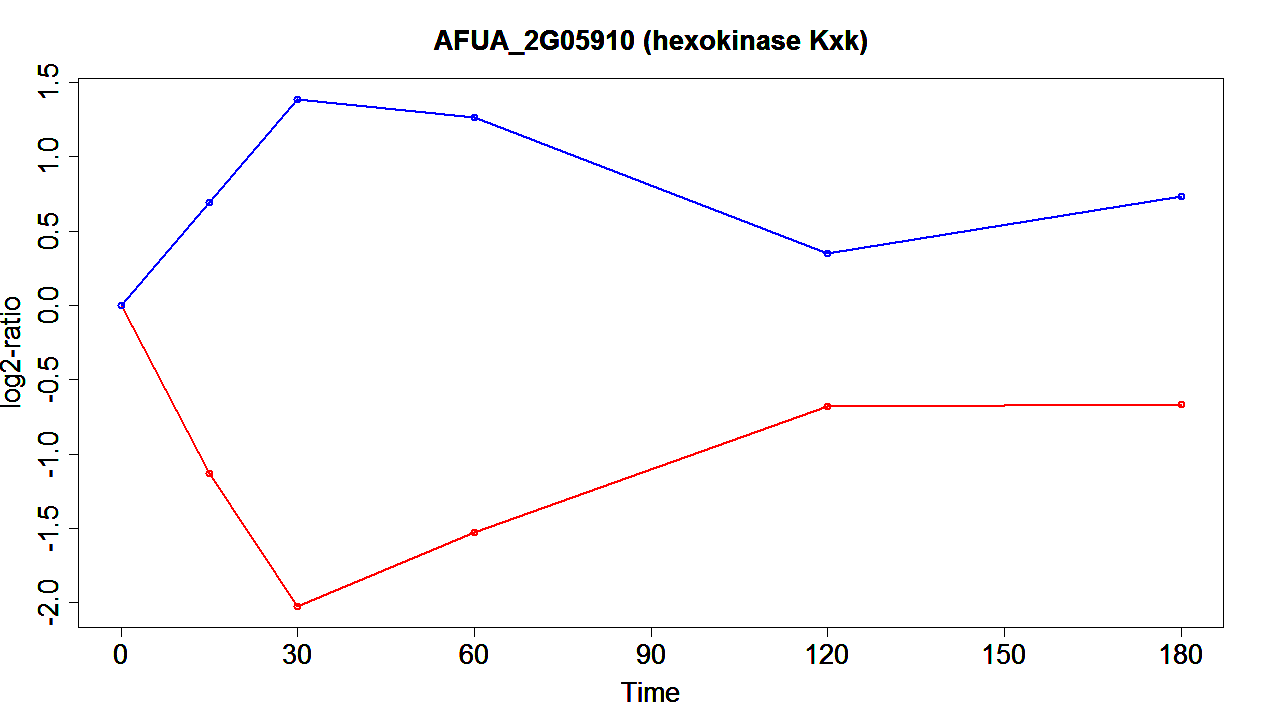  **Time series of differentially regulated transcripts and respective proteins**  **Relative abundance of protein spots in log_2_ ratios**  **Time in minutes after temperature shift**  **Red – transcript, blue – protein** |
| 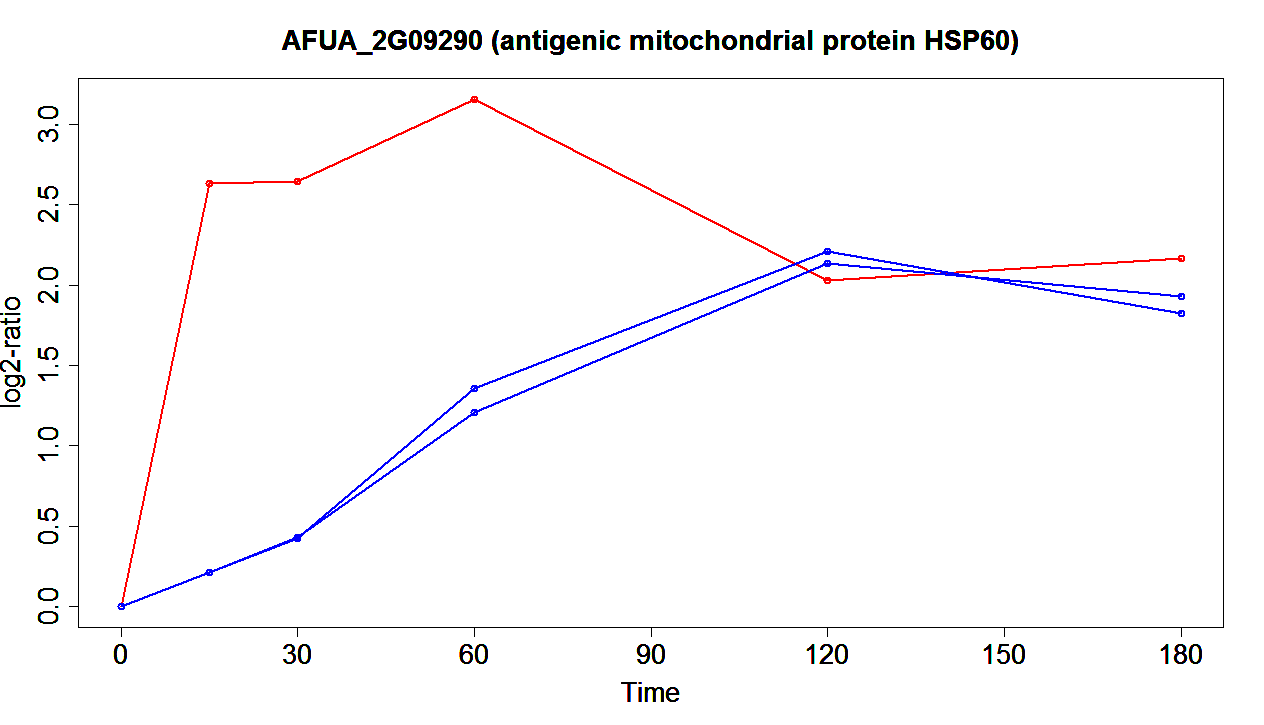  **Time series of differentially regulated transcripts and respective proteins**  **Relative abundance of protein spots in log_2_ ratios**  **Time in minutes after temperature shift**  **Red – transcript, blue – protein**  **several blue lines indicate several spots representing the same protein** |
| 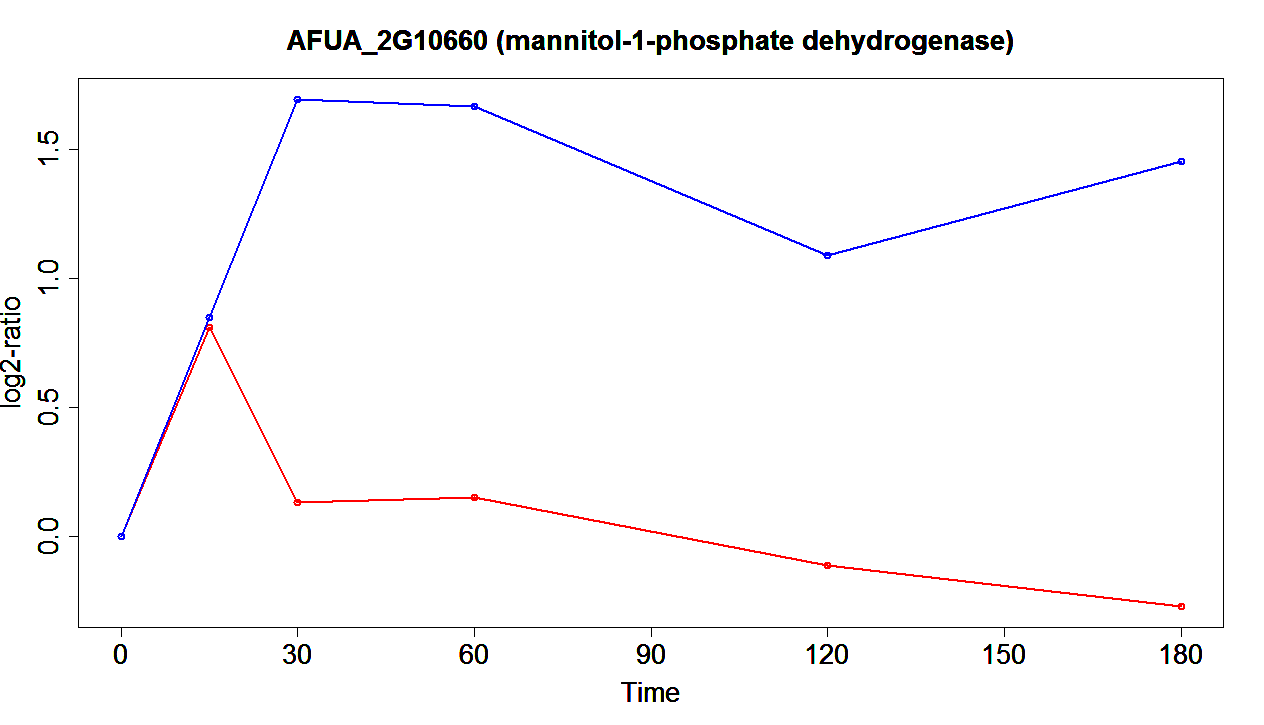  **Time series of differentially regulated transcripts and respective proteins**  **Relative abundance of protein spots in log_2_ ratios**  **Time in minutes after temperature shift**  **Red – transcript, blue – protein** |
| 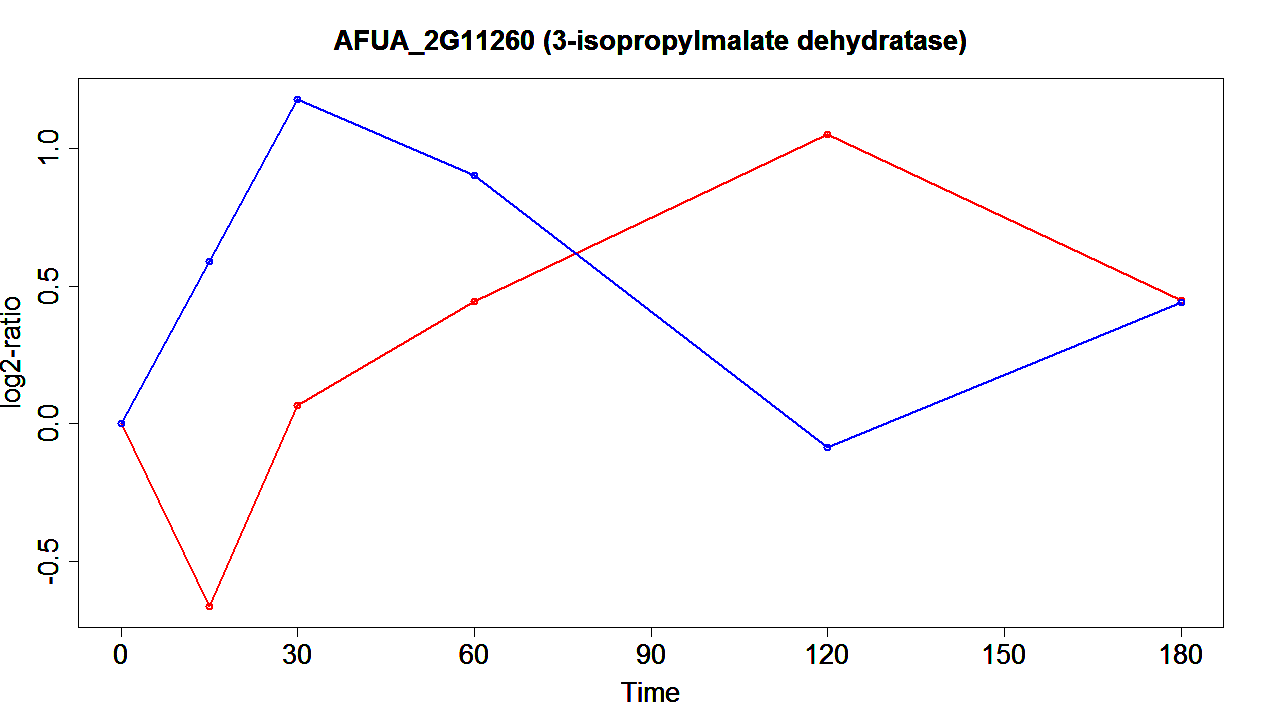  **Time series of differentially regulated transcripts and respective proteins**  **Relative abundance of protein spots in log_2_ ratios**  **Time in minutes after temperature shift**  **Red – transcript, blue – protein** |
| 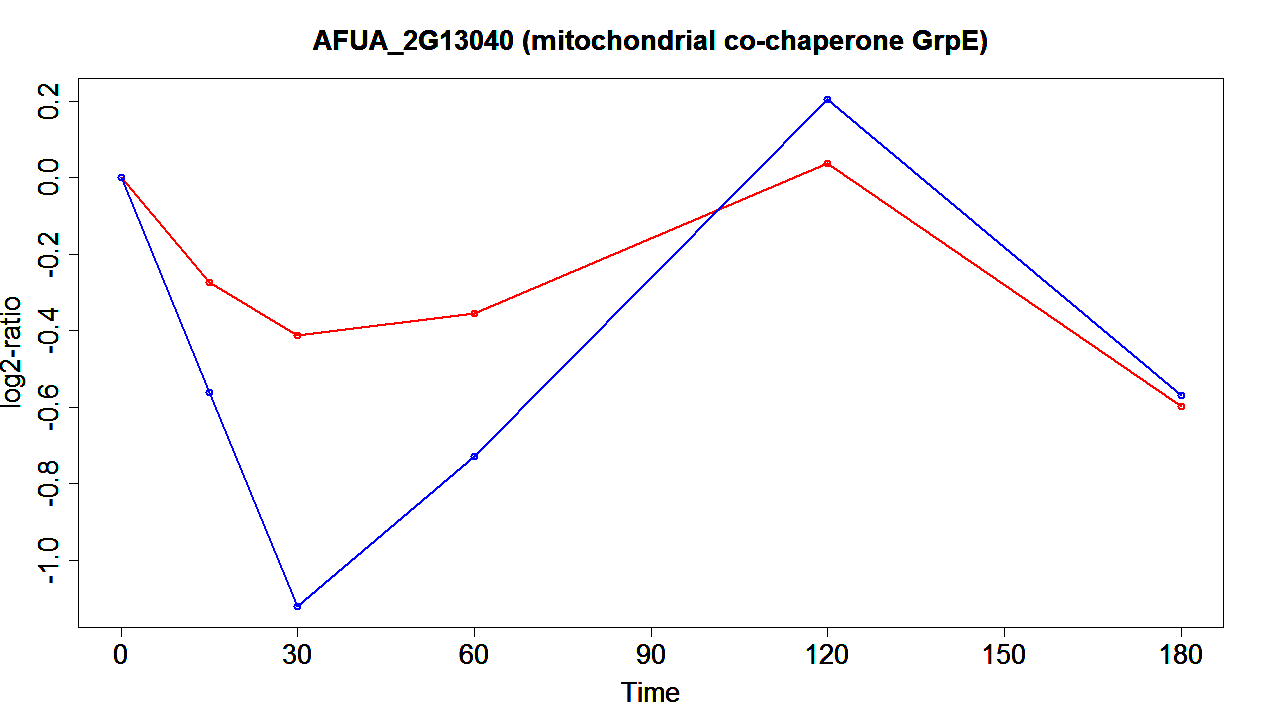  **Time series of differentially regulated transcripts and respective proteins**  **Relative abundance of protein spots in log_2_ ratios**  **Time in minutes after temperature shift**  **Red – transcript, blue – protein** |
| 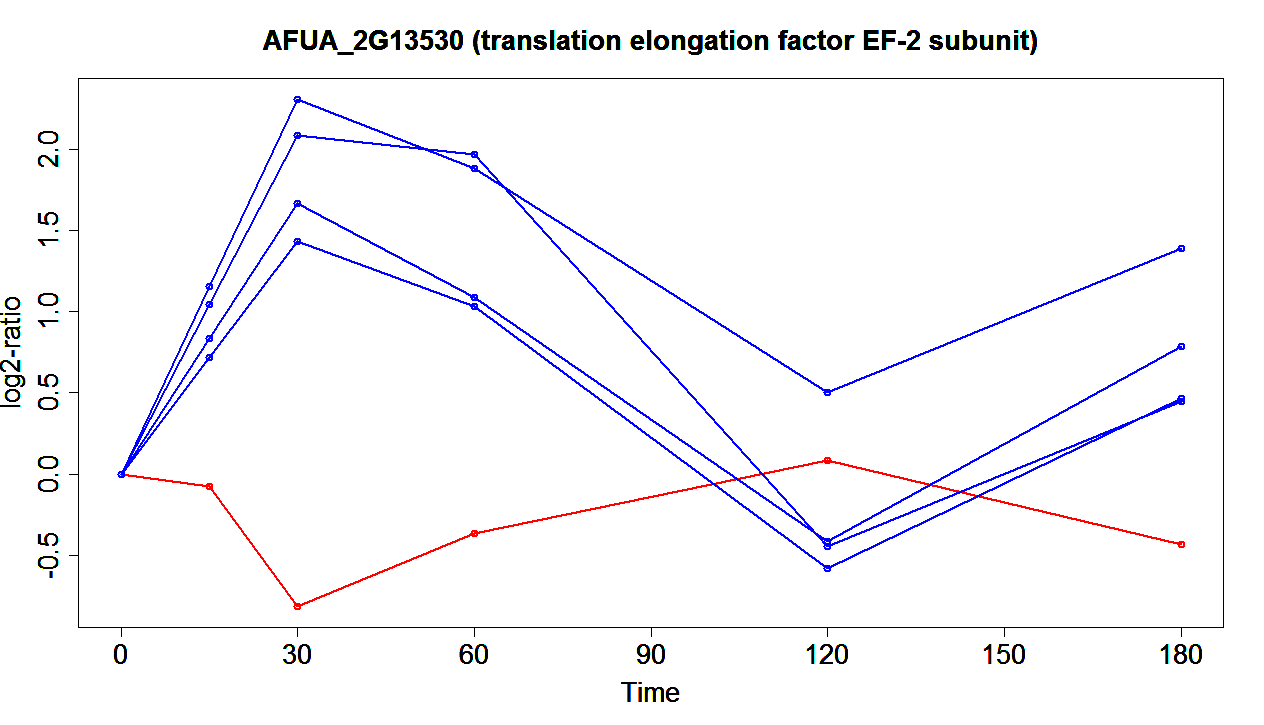  **Time series of differentially regulated transcripts and respective proteins**  **Relative abundance of protein spots in log_2_ ratios**  **Time in minutes after temperature shift**  **Red – transcript, blue – protein**  **several blue lines indicate several spots representing the same protein** |
| 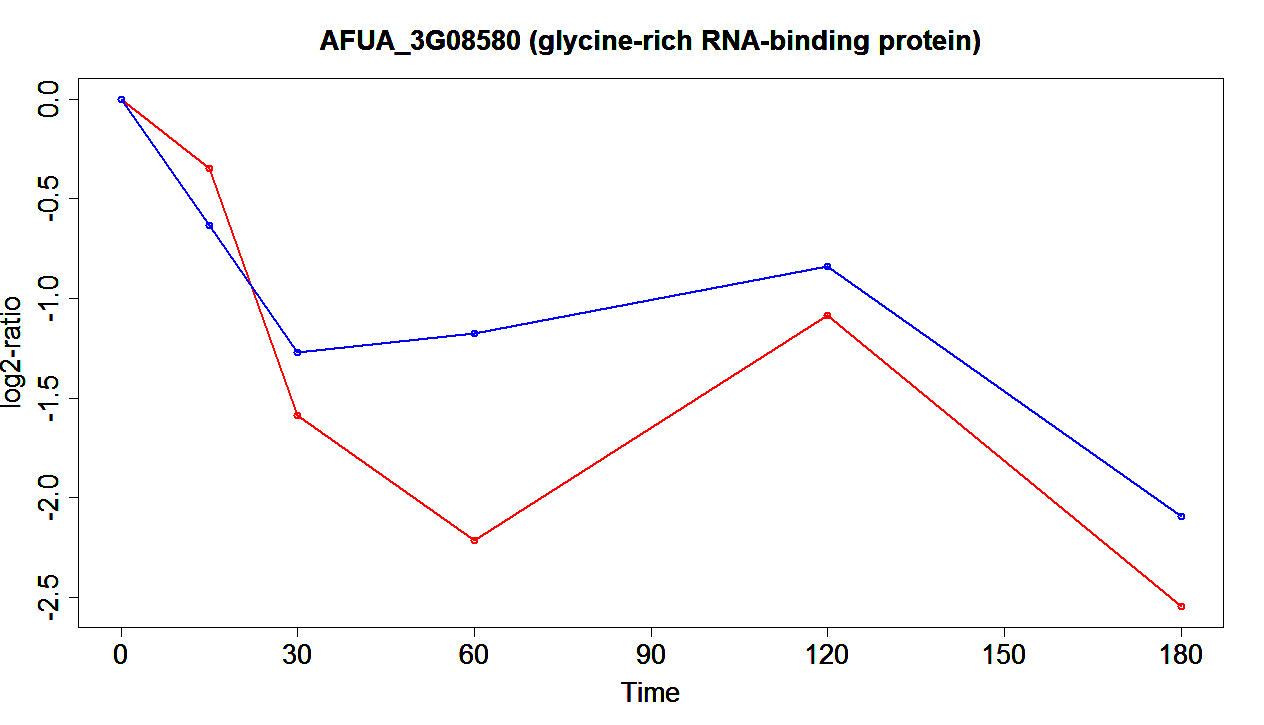  **Time series of differentially regulated transcripts and respective proteins**  **Relative abundance of protein spots in log_2_ ratios**  **Time in minutes after temperature shift**  **Red – transcript, blue – protein** |
| 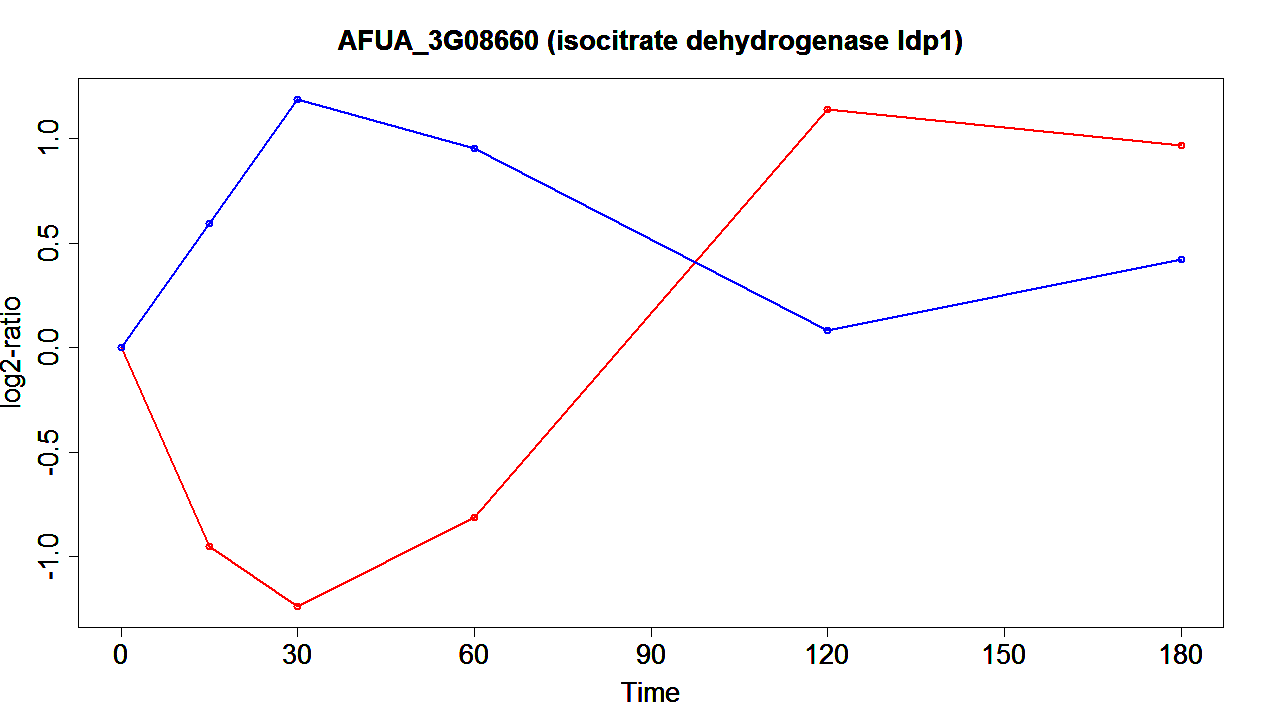  **Time series of differentially regulated transcripts and respective proteins**  **Relative abundance of protein spots in log_2_ ratios**  **Time in minutes after temperature shift**  **Red – transcript, blue – protein** |
| 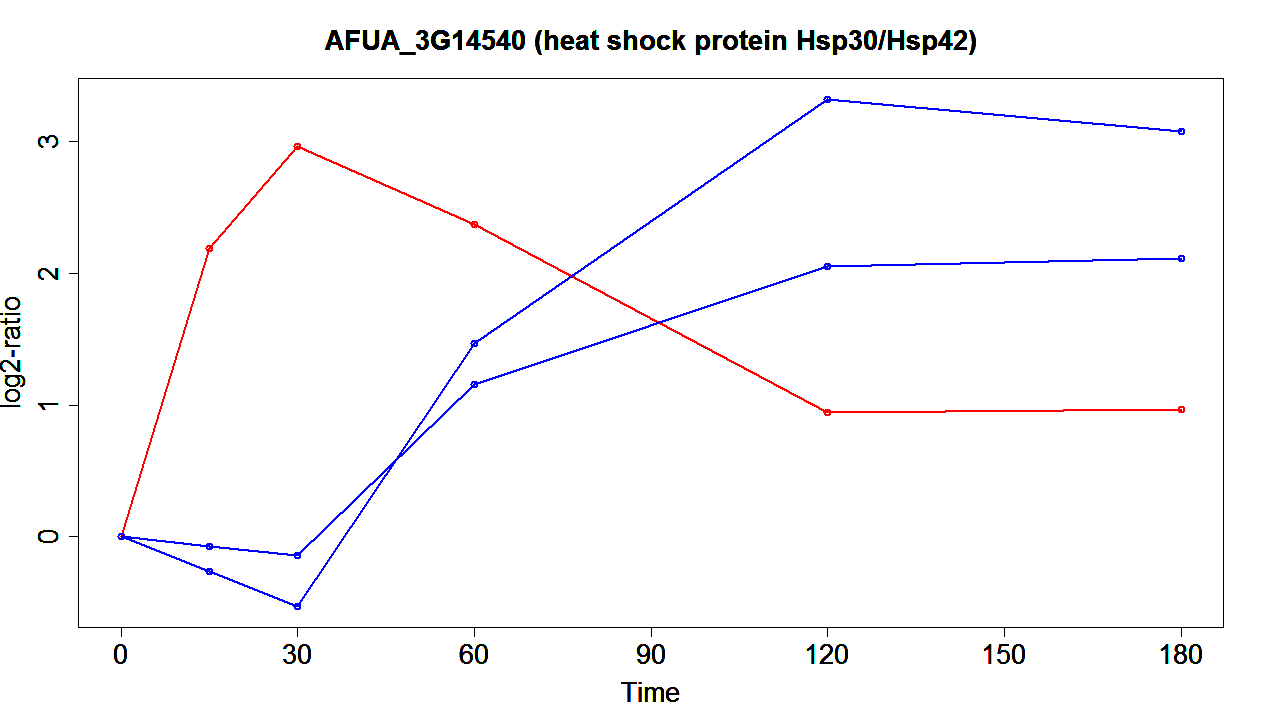  **Time series of differentially regulated transcripts and respective proteins**  **Relative abundance of protein spots in log_2_ ratios**  **Time in minutes after temperature shift**  **Red – transcript, blue – protein**  **several blue lines indicate several spots representing the same protein** |
| 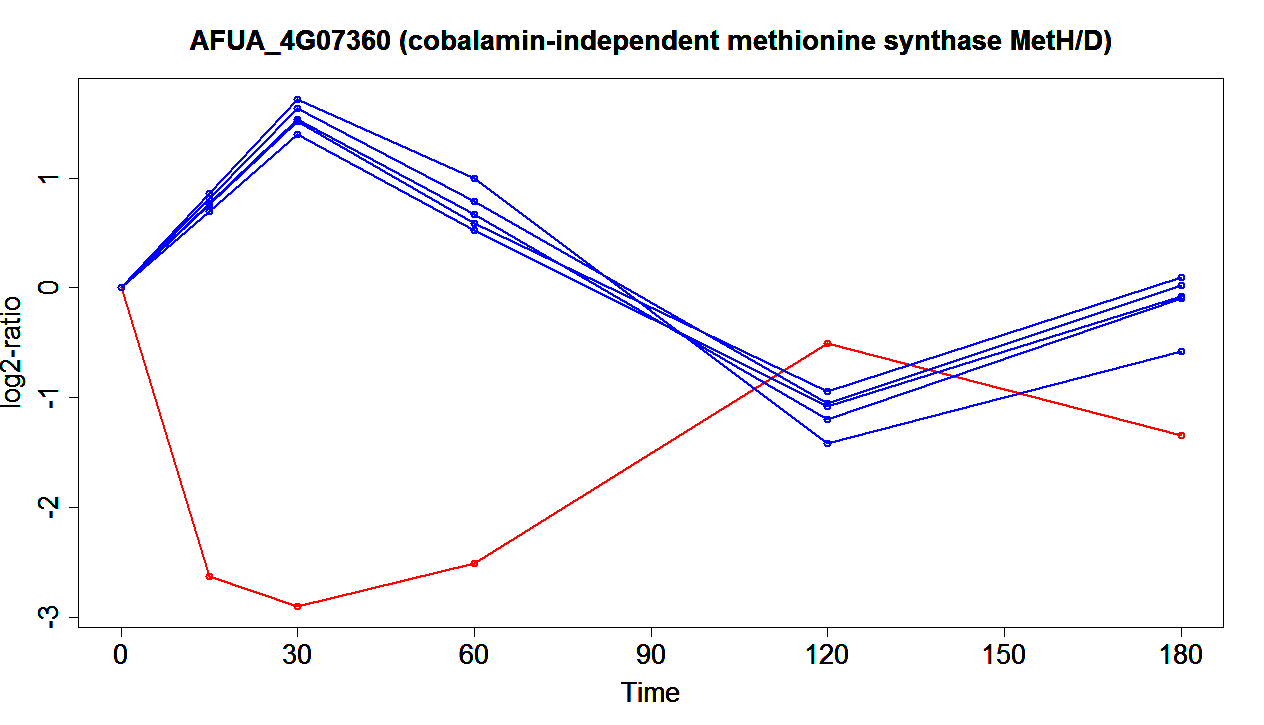  **Time series of differentially regulated transcripts and respective proteins**  **Relative abundance of protein spots in log_2_ ratios**  **Time in minutes after temperature shift**  **Red – transcript, blue – protein**  **several blue lines indicate several spots representing the same protein** |
| 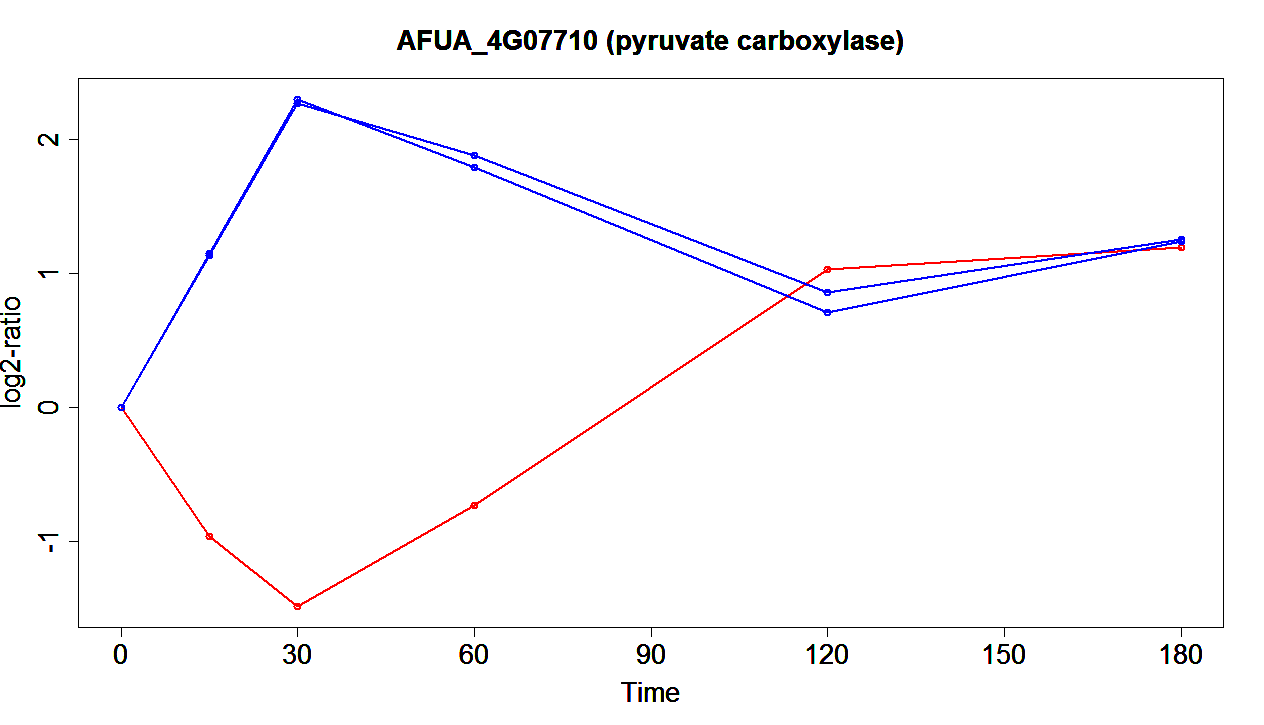  **Time series of differentially regulated transcripts and respective proteins**  **Relative abundance of protein spots in log_2_ ratios**  **Time in minutes after temperature shift**  **Red – transcript, blue – protein**  **several blue lines indicate several spots representing the same protein** |
| 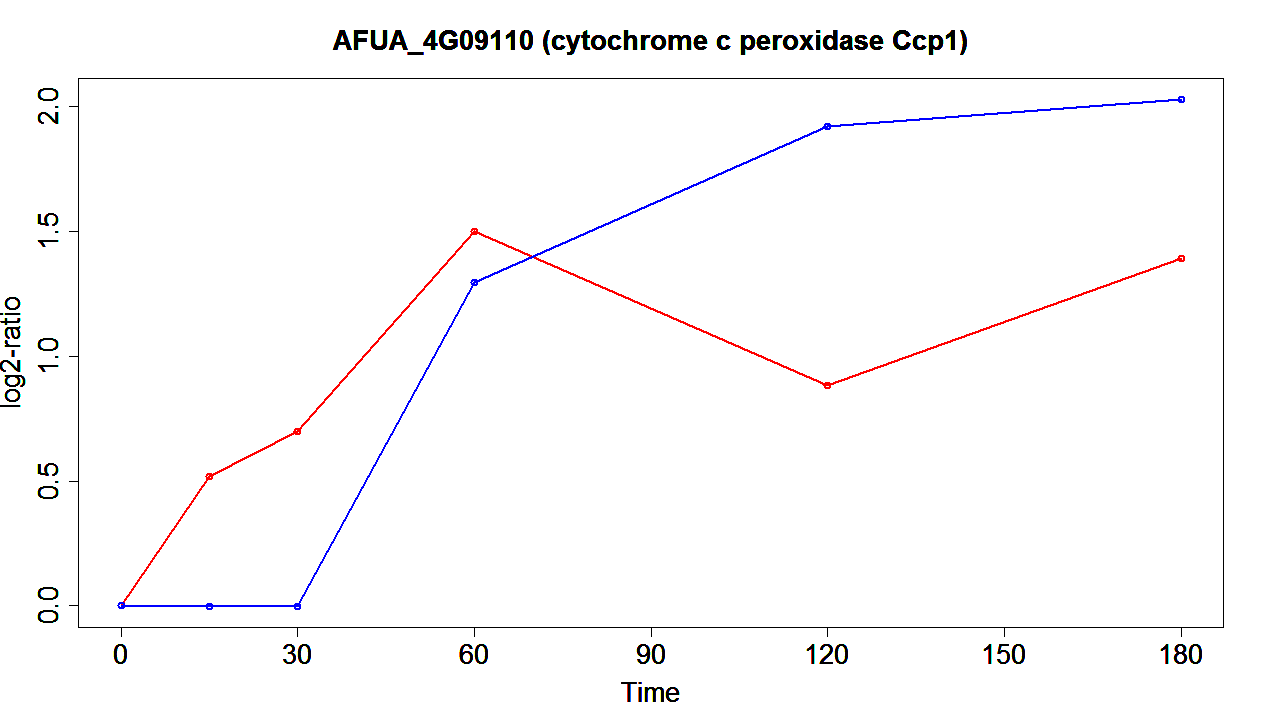  **Time series of differentially regulated transcripts and respective proteins**  **Relative abundance of protein spots in log_2_ ratios**  **Time in minutes after temperature shift**  **Red – transcript, blue – protein** |
| 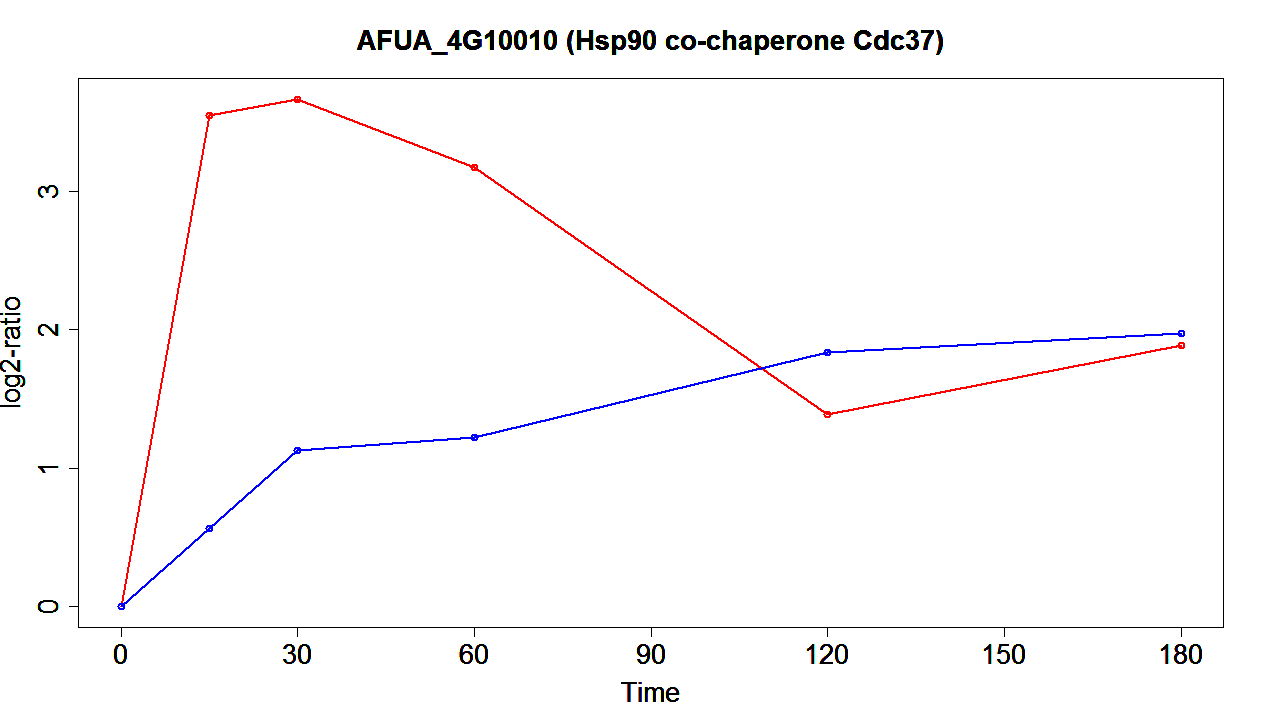  **Time series of differentially regulated transcripts and respective proteins**  **Relative abundance of protein spots in log_2_ ratios**  **Time in minutes after temperature shift**  **Red – transcript, blue – protein** |
| 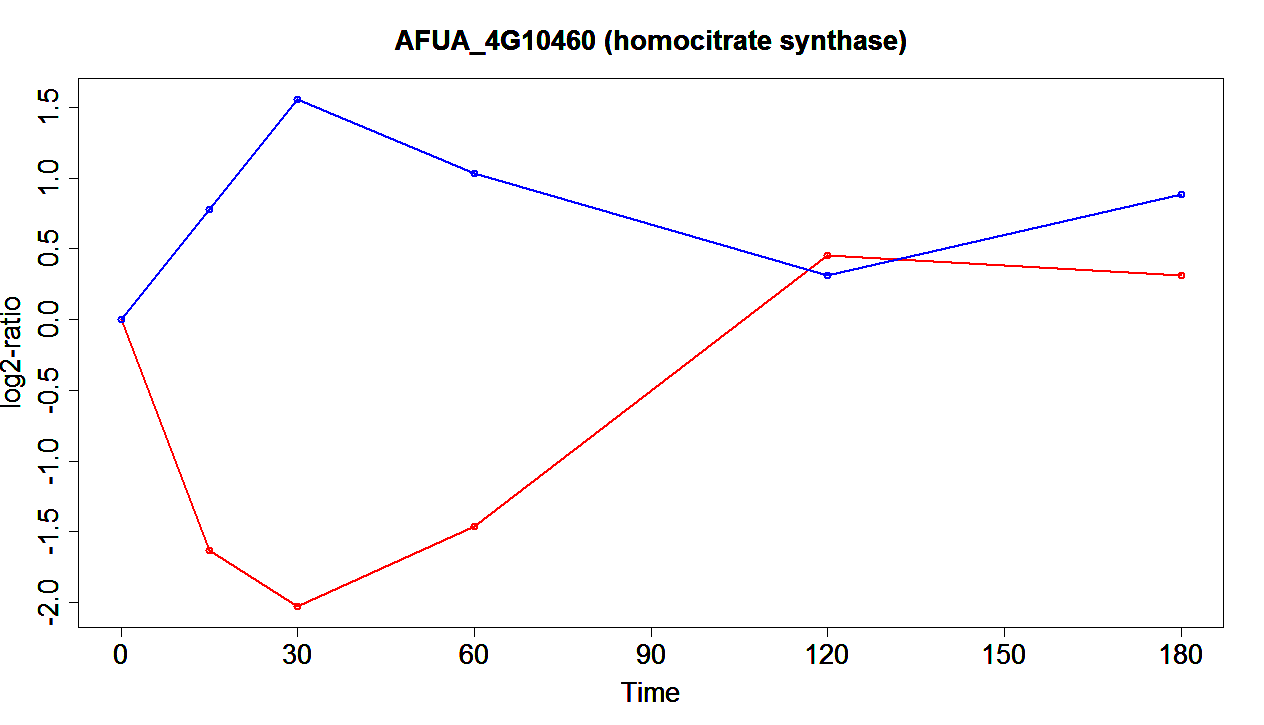  **Time series of differentially regulated transcripts and respective proteins**  **Relative abundance of protein spots in log_2_ ratios**  **Time in minutes after temperature shift**  **Red – transcript, blue – protein** |
| 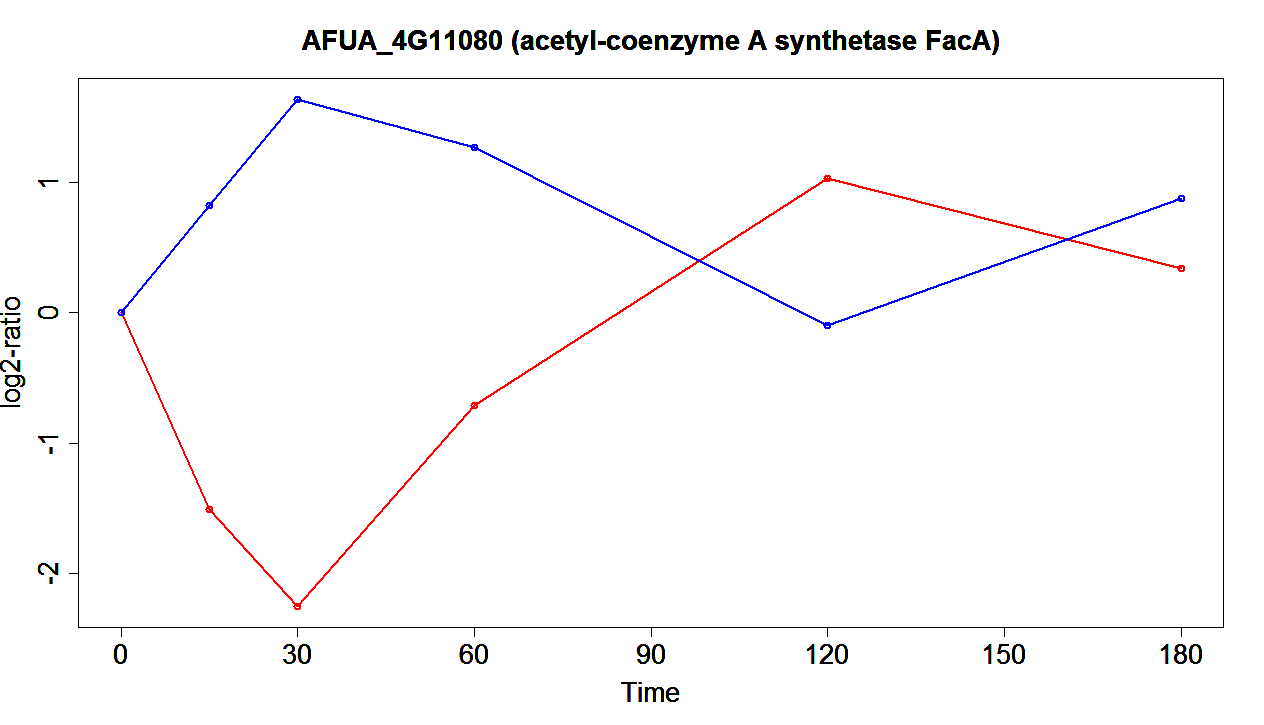  **Time series of differentially regulated transcripts and respective proteins**  **Relative abundance of protein spots in log_2_ ratios**  **Time in minutes after temperature shift**  **Red – transcript, blue – protein** |
| 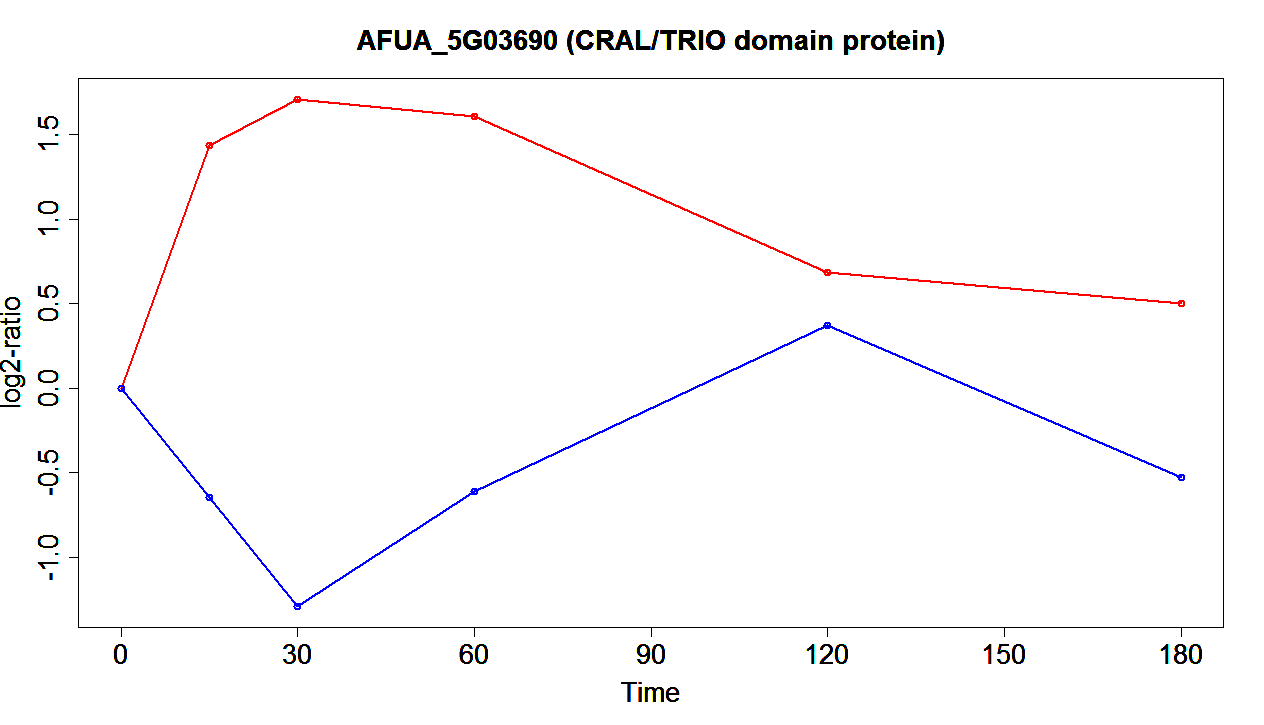  **Time series of differentially regulated transcripts and respective proteins**  **Relative abundance of protein spots in log_2_ ratios**  **Time in minutes after temperature shift**  **Red – transcript, blue – protein** |
| 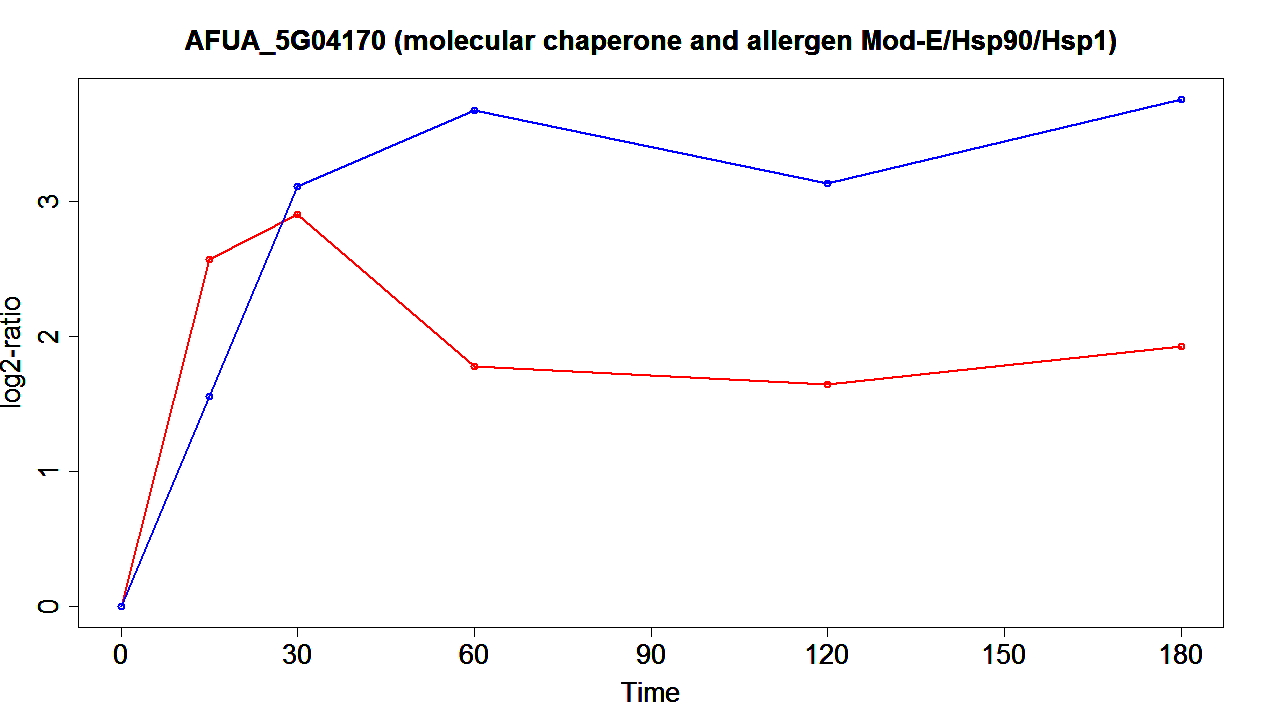  **Time series of differentially regulated transcripts and respective proteins**  **Relative abundance of protein spots in log_2_ ratios**  **Time in minutes after temperature shift**  **Red – transcript, blue – protein** |
| 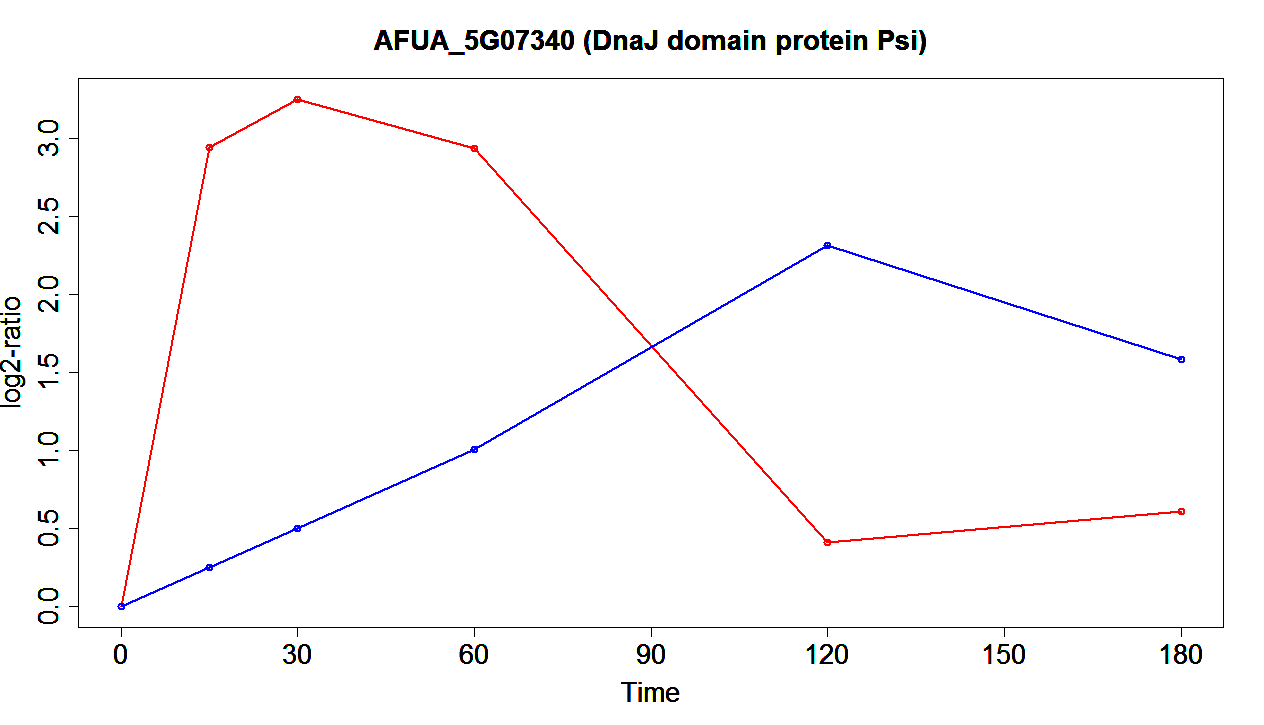  **Time series of differentially regulated transcripts and respective proteins**  **Relative abundance of protein spots in log_2_ ratios**  **Time in minutes after temperature shift**  **Red – transcript, blue – protein** |
| 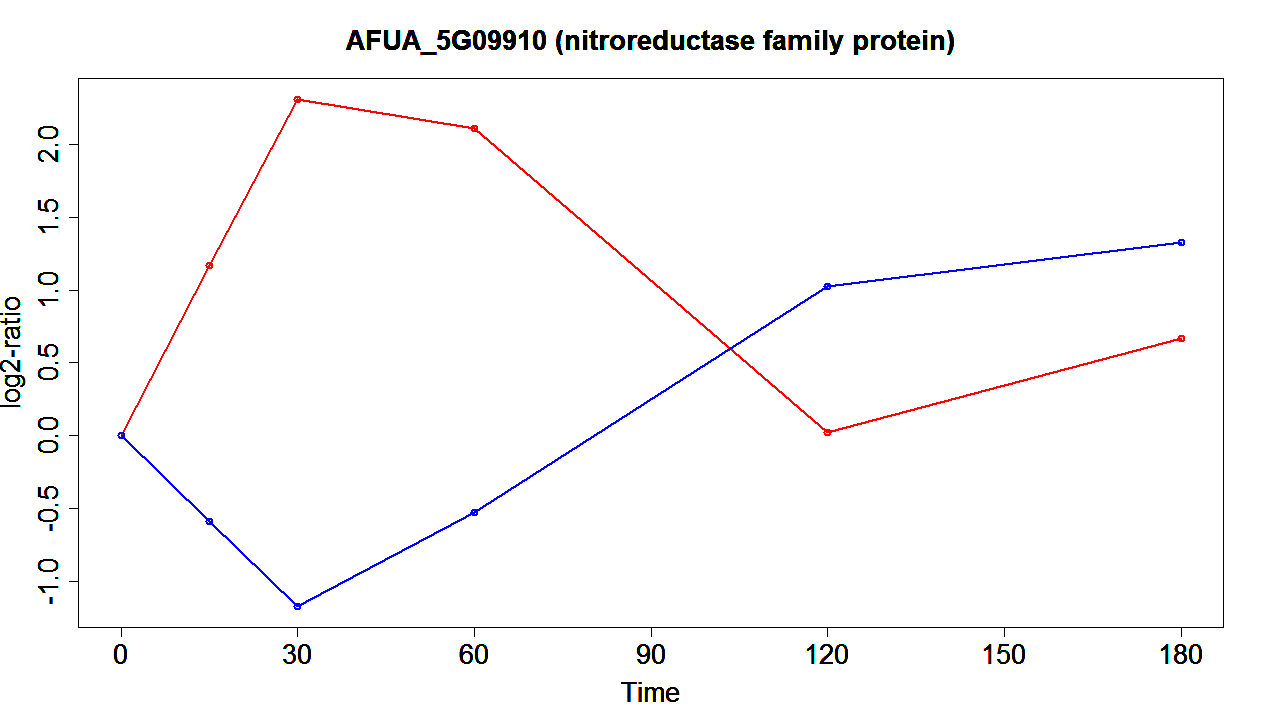  **Time series of differentially regulated transcripts and respective proteins**  **Relative abundance of protein spots in log_2_ ratios**  **Time in minutes after temperature shift**  **Red – transcript, blue – protein** |
| 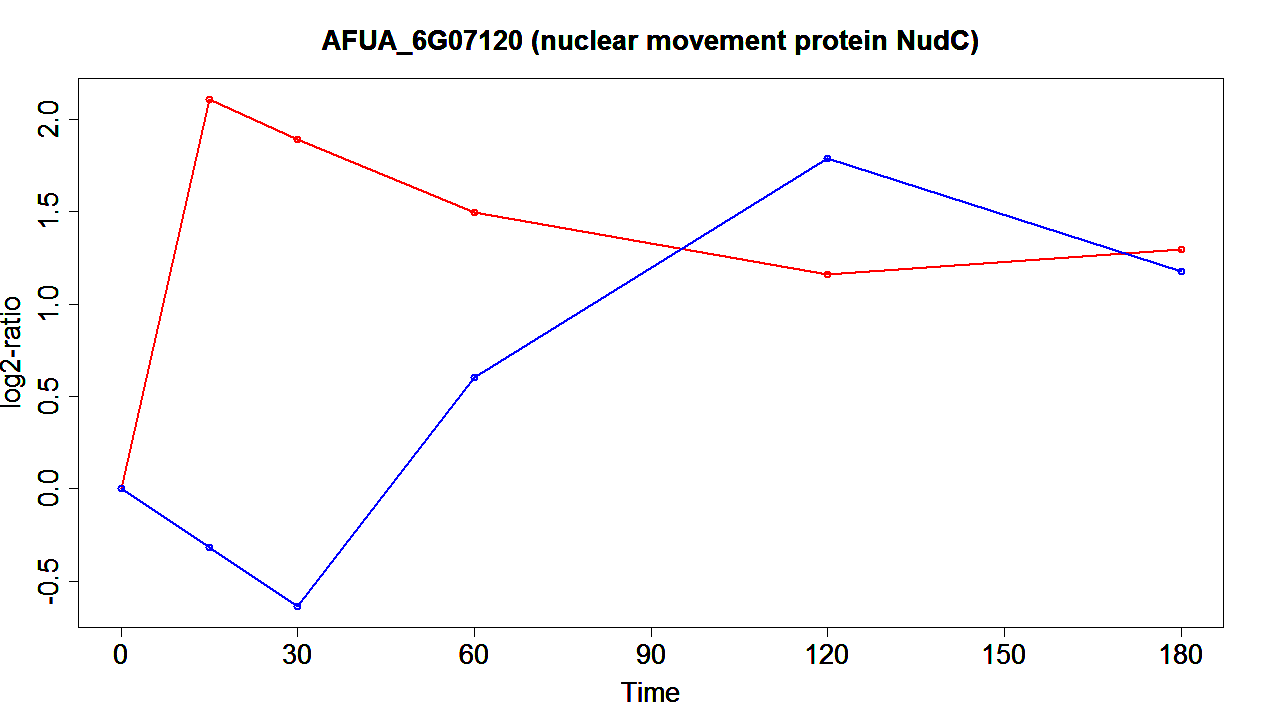  **Time series of differentially regulated transcripts and respective proteins**  **Relative abundance of protein spots in log_2_ ratios**  **Time in minutes after temperature shift**  **Red – transcript, blue – protein** |
| 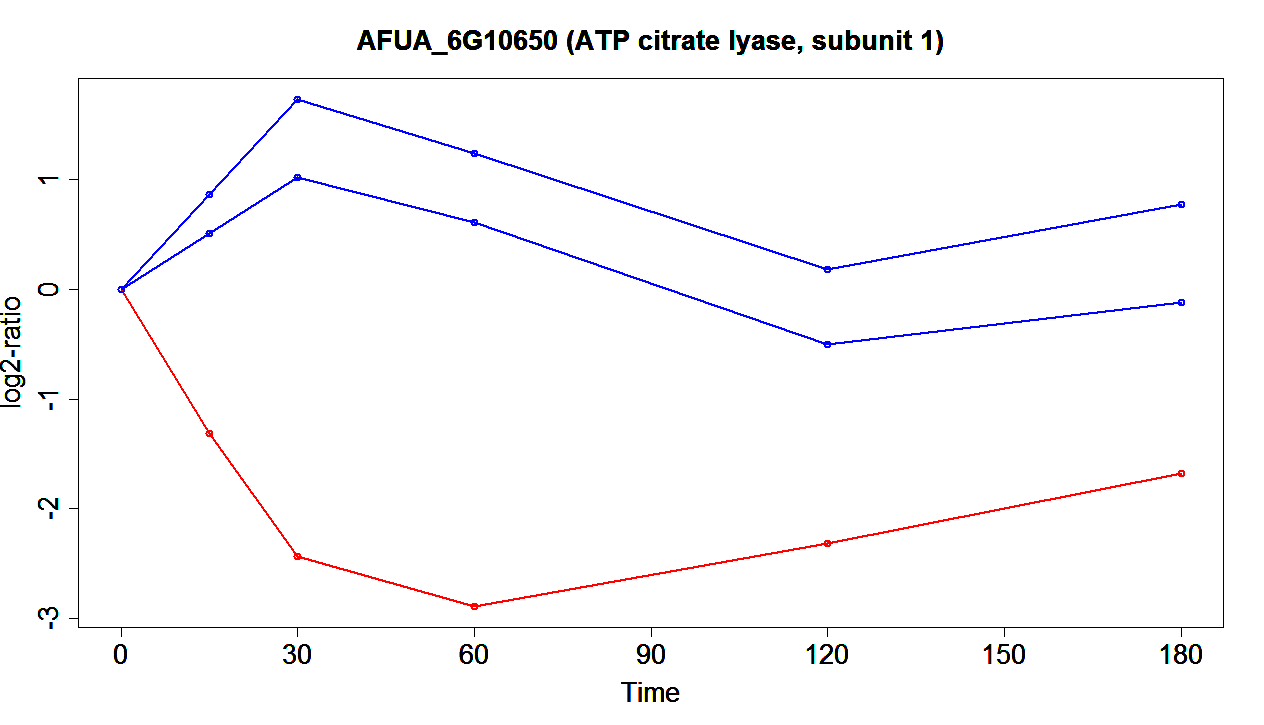  **Time series of differentially regulated transcripts and respective proteins**  **Relative abundance of protein spots in log_2_ ratios**  **Time in minutes after temperature shift**  **Red – transcript, blue – protein**  **several blue lines indicate several spots representing the same protein** |
| 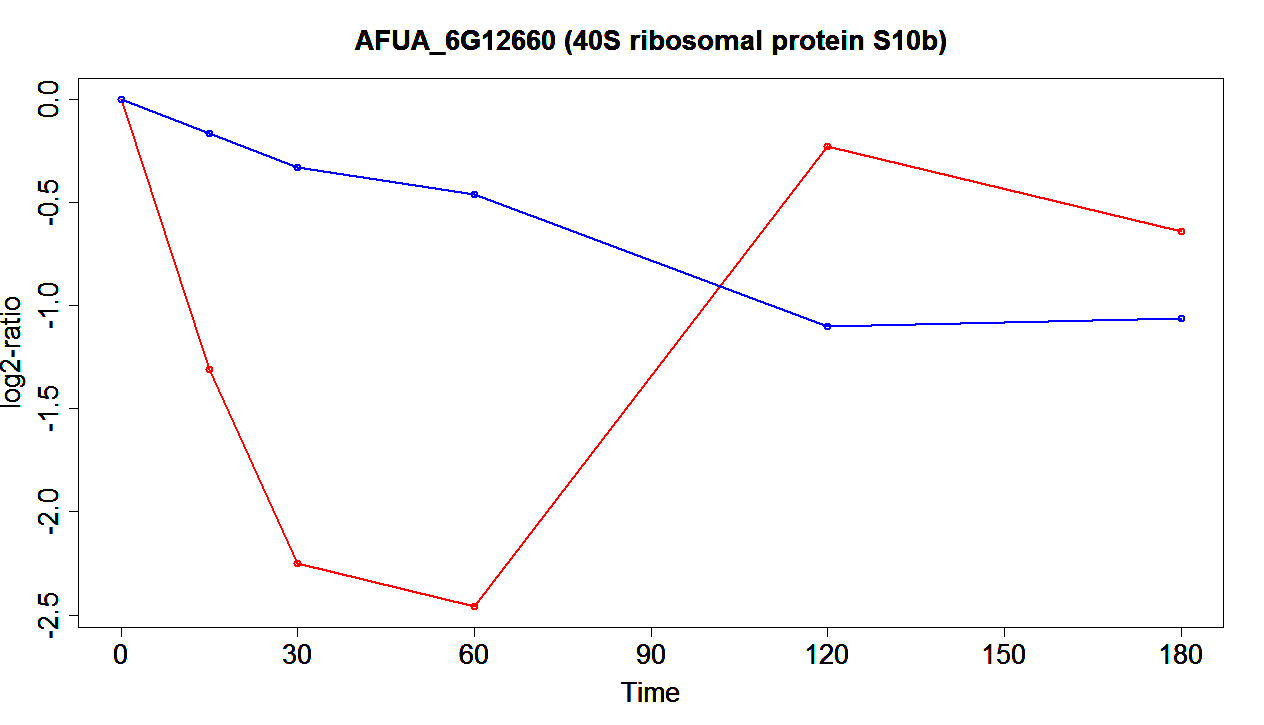  **Time series of differentially regulated transcripts and respective proteins**  **Relative abundance of protein spots in log_2_ ratios**  **Time in minutes after temperature shift**  **Red – transcript, blue – protein** |
| 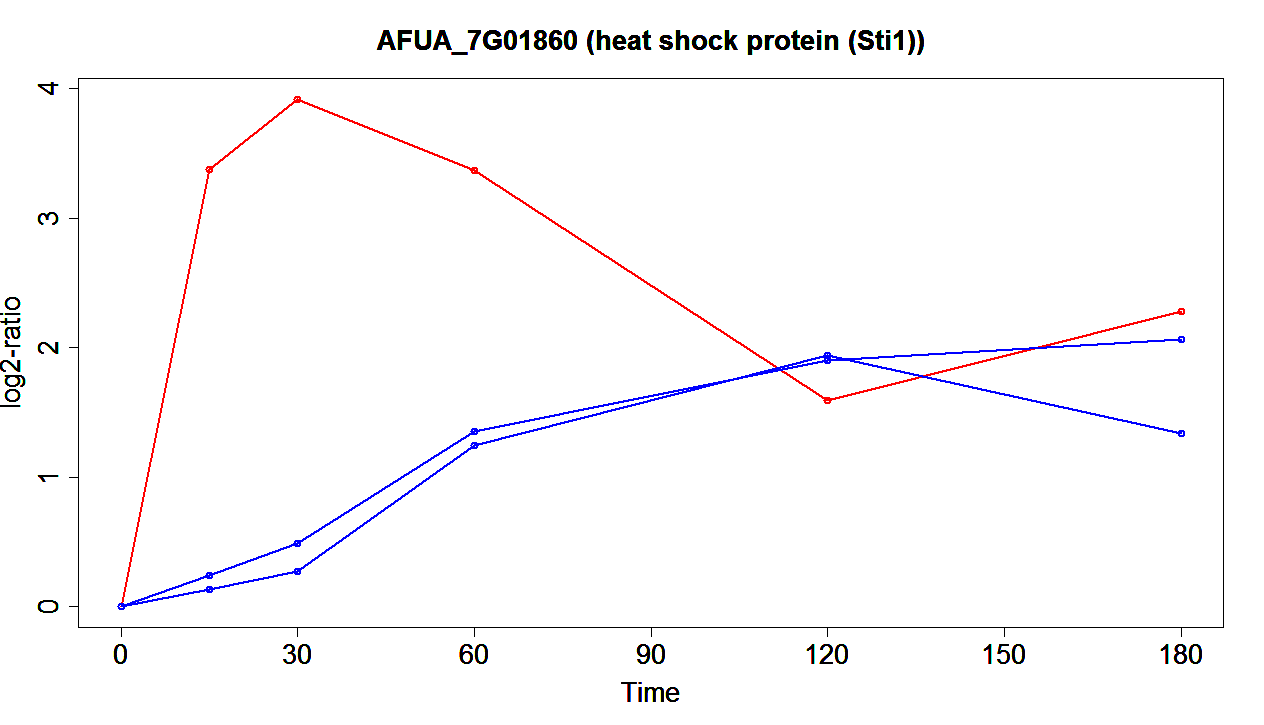  **Time series of differentially regulated transcripts and respective proteins**  **Relative abundance of protein spots in log_2_ ratios**  **Time in minutes after temperature shift**  **Red – transcript, blue – protein**  **several blue lines indicate several spots representing the same protein** |
| 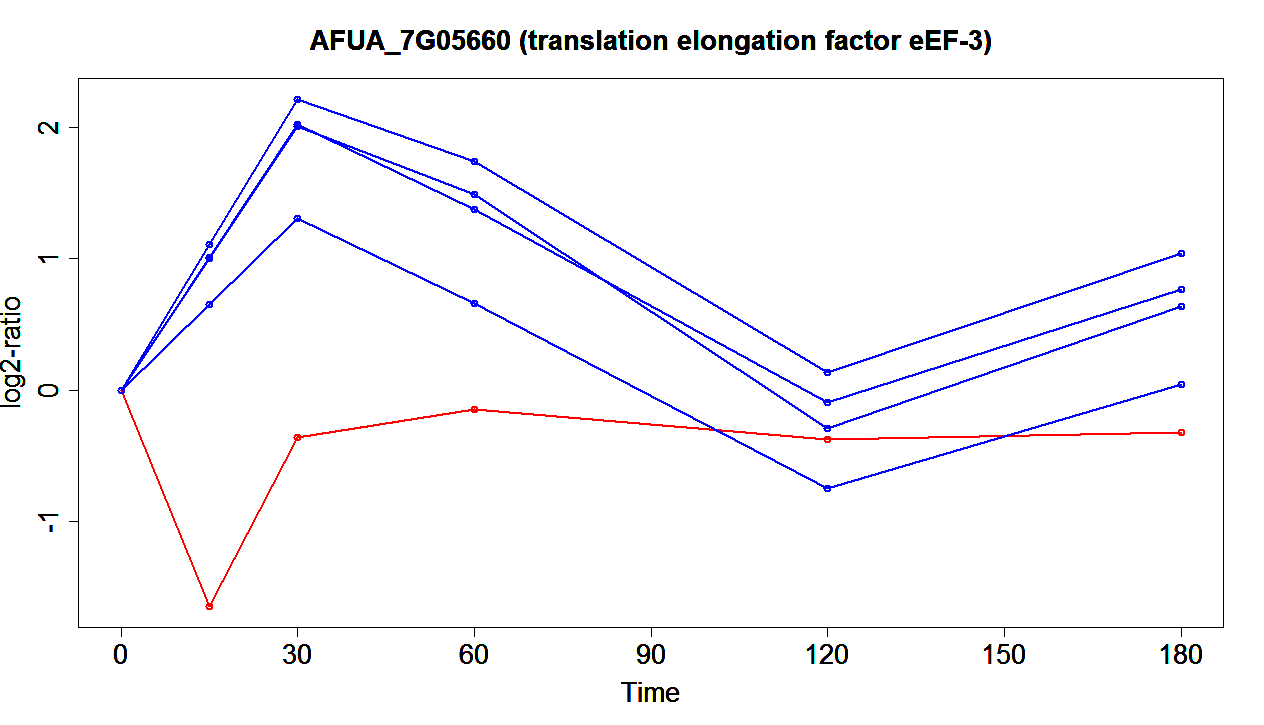  **Time series of differentially regulated transcripts and respective proteins**  **Relative abundance of protein spots in log_2_ ratios**  **Time in minutes after temperature shift**  **Red – transcript, blue – protein**  **several blue lines indicate several spots representing the same protein** |
