# Supplementary figures and images for: Integrative analysis of the heat shock response in Aspergillus fumigatus
Source: BMC Genomics. 2010 Jan 15;11:32. doi: 10.1186/1471-2164-11-32 (PMC2820008; doi:10.1186/1471-2164-11-32)

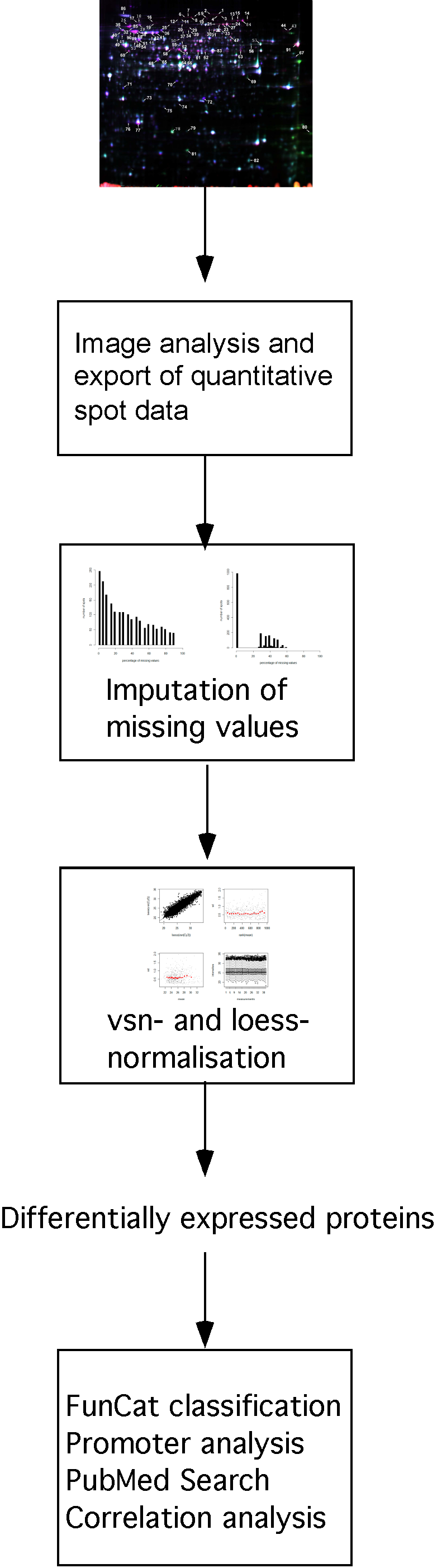

Supplement: Additional file 5 — Workflow. The DIGE analysis workflow of this study. [file 1471-2164-11-32-S5.TIFF]
